# Neuropeptide Y/Y5 Receptor Pathway Stimulates Neuroblastoma Cell Motility Through RhoA Activation

**DOI:** 10.3389/fcell.2020.627090

**Published:** 2021-02-17

**Authors:** Nouran Abualsaud, Lindsay Caprio, Susana Galli, Ewa Krawczyk, Lamia Alamri, Shiya Zhu, G. Ian Gallicano, Joanna Kitlinska

**Affiliations:** ^1^Department of Biochemistry and Molecular and Cellular Biology, Georgetown University, Washington, DC, United States; ^2^Cell Therapy and Cancer Research Department, King Abdullah International Medical Research Center, Riyadh, Saudi Arabia; ^3^King Saud bin Abdulaziz University for Health Sciences, Riyadh, Saudi Arabia; ^4^Department of Human Science, School of Nursing and Health Studies, Georgetown University, Washington, DC, United States; ^5^Center for Cell Reprogramming, Georgetown University Medical Center, Washington, DC, United States; ^6^Department of Obstetrics and Gynecology, Vanderbilt University Medical Center, Nashville, TN, United States

**Keywords:** neuropeptide Y, neuropeptide Y receptor Y5, neuroblastoma, cell migration, RhoA

## Abstract

Neuropeptide Y (NPY) has been implicated in the regulation of cellular motility under various physiological and pathological conditions, including cancer dissemination. Yet, the exact signaling pathways leading to these effects remain unknown. In a pediatric malignancy, neuroblastoma (NB), high NPY release from tumor tissue associates with metastatic disease. Here, we have shown that NPY stimulates NB cell motility and invasiveness and acts as a chemotactic factor for NB cells. We have also identified the Y5 receptor (Y5R) as the main NPY receptor mediating these actions. In NB tissues and cell cultures, Y5R is highly expressed in migratory cells and accumulates in regions of high RhoA activity and dynamic cytoskeleton remodeling. Y5R stimulation activates RhoA and results in Y5R/RhoA-GTP interactions, as shown by pull-down and proximity ligation assays, respectively. This is the first demonstration of the role for the NPY/Y5R axis in RhoA activation and the subsequent cytoskeleton remodeling facilitating cell movement. These findings implicate Y5R as a target in anti-metastatic therapies for NB and other cancers expressing this receptor.

## Introduction

Neuroblastoma (NB) is a pediatric malignancy arising due to defects in sympathetic neuron differentiation (Maris, 2010; Matthay et al., 2016). The disease is heterogenous. Approximately half of NB patients present with high-risk metastatic disease at diagnosis, which associates with a 40–50% 5-year survival rate (Cohn et al., 2009). A large group of NB patients does not respond to treatment, which often leads to the secondary disease dissemination (Maris, 2010; Smith and Foster, 2018). It has been suggested that the presence of a chemoresistant NB cell population with a cancer stem cell phenotype drives metastasis and poor clinical outcomes in high-risk NB (Maris, 2010; Bahmad et al., 2019). Thus, targeting this NB cell sub-population is critical in the treatment of the refractory disease.

Due to their sympathetic origin, NBs express neuronal markers, such as neuropeptide Y (NPY) (Kitlinska et al., 2005; Galli et al., 2016). NPY is a neurotransmitter normally released from mature sympathetic nerves, acting via multiple G-protein-coupled receptors (GPCRs), termed Y1, Y2, and Y5 receptors (Y1R, Y2R, and Y5R, respectively) (Zukowska-Grojec, 1995). In addition to its numerous physiological functions, NPY exhibits pleotropic effects relevant to tumor biology, such as stimulation of angiogenesis, cell proliferation, migration, chemoresistance, and regulation of stem cell differentiation (Tilan and Kitlinska, 2016; Yi et al., 2017).

NB cells constitutively express NPY and Y2R, creating an autocrine loop, which maintains their proliferation (Kitlinska et al., 2005; Lu et al., 2010; Galli et al., 2016). Moreover, Y2R expressed in endothelial cells mediates the angiogenic effect of NPY and thereby contributes to tumor vascularization (Lee et al., 2003; Kitlinska et al., 2005; Lu et al., 2010). Consequently, in animal models, the Y2R antagonist inhibits the growth of NB xenografts due to its anti-proliferative and anti-angiogenic effects (Lu et al., 2010). In contrast, Y5R expression is induced in NB cells under pro-apoptotic conditions and promotes their survival (Czarnecka et al., 2015). Consequently, the expression of both NPY and Y5R is elevated in chemoresistant NBs and cell lines developed from recurrent tumors, while Y5R antagonist inhibits NB tumor growth via pro-apoptotic effects (Czarnecka et al., 2015).

In addition to the effect of NPY on NB growth and vascularization, growing evidence indicates that the peptide may also be involved in the dissemination of the disease. In NB patients, elevated serum NPY levels correlate with metastases, with the median NPY concentrations of 0.44 and 1.11 ng/ml in patients with localized disease and distant metastases, respectively (Galli et al., 2016). High circulating NPY associates also with worse survival (Galli et al., 2016). The 5-year overall survival rates are at 58% for patients with elevated serum NPY, compared to 87% in those with its normal levels. In NB tissues at diagnosis, Y5R is highly expressed in tumor cells with an angioinvasive phenotype (Galli et al., 2016). A similar pattern of Y5R expression was observed in an animal model of NB, where high expression of both Y5R and NPY marked a specific population of angioinvasive NB cells in primary tumors, while all cells within newly formed metastases were positive for these proteins (Galli et al., 2016). Altogether, these data suggested a role for the NPY/Y5R axis in local NB cell invasiveness and distant metastasis. Similarly, NPY and its Y5R have been implicated in the motility of Ewing sarcoma, breast, and liver cancer cells (Medeiros et al., 2011; Tilan et al., 2013; Dietrich et al., 2020). The peptide also promotes the migration of non-tumoral cells, e.g., endothelial cells during angiogenesis (Movafagh et al., 2006). However, the mechanisms underlying these effects remain unknown. Importantly, many NPY functions, including cell proliferation and migration, are enhanced by interactions of its heterotypic receptors, which enable a cellular response to its low concentrations (Movafagh et al., 2006; Pons et al., 2008; Czarnecka et al., 2019). Such interactions involve either receptor heterodimerization, as shown for Y1R and Y5R, or indirect cross-talk between signaling pathways of different receptors, including Y2R and Y5R (Gehlert et al., 2007; Kilpatrick et al., 2015; Czarnecka et al., 2019).

Cell migration, an essential feature facilitating cancer dissemination, is associated with morphological alterations driven by dynamic cytoskeleton remodeling (Friedl, 2004; Huttenlocher and Horwitz, 2011). Single migratory cells are characterized by their bi-polar phenotype. At the leading edge, actin polymerization into its filamentous form (F-actin) leads to development of protrusions, such as lamellipodia and filopodia, which facilitate forward movement of the cells (Lintz et al., 2017). Cytoskeleton changes in the trailing edge involve actin–myosin interactions responsible for cell tail retraction (Lintz et al., 2017). Cancer cells can also migrate collectively, which has been associated with their aggressive phenotype (Lintz et al., 2017). During collective migration, the stable cell–cell junctions that are present in the established tissues are replaced by dynamic junctions, which maintain cell unity, while enabling their movement as sheets, strands, or clusters (Friedl and Mayor, 2017; Lintz et al., 2017). The migration of these collective units is driven by leader cells that have unique molecular characteristics (Reffay et al., 2014; Lintz et al., 2017).

RhoA, a small protein belonging to the Rho family of GTPases, is one of the key cytoskeleton regulators (O'connor and Chen, 2013). Its activity is tightly controlled in a spatiotemporal manner during cell migration (Bolado-Carrancio et al., 2020). In single migrating cells, RhoA is responsible for actin polymerization at the leading edge, while at the trailing edge, it controls actomyosin contractility and cell tail retraction (Narumiya et al., 2009; Ridley, 2015). In the context of collective migration, RhoA activity is particularly high in leader cells, facilitating development of the functional leading edge (Zegers and Friedl, 2014). At the supracellular level, RhoA is involved in formation of the actomyosin cables spanning along the outer edge of the multicellular migration unit and maintaining its integrity (Zegers and Friedl, 2014). Last, RhoA activity is essential to preserve cell–cell junctions during collective migration (Theveneau and Mayor, 2010; Zegers and Friedl, 2014). However, overactivation of the RhoA pathway may lead to excessive stress fiber formation and inhibition of cell migration (Ridley, 2015). Hence, the precise regulation of the time, localization and extent of RhoA activity are essential for cell movement.

The goal of the current study was to determine the role of the NPY system in NB cell migration and identify the mechanisms of its action. Using both NB cells and CHO-K1 cells transfected with single types of NPY receptors, we have provided the first evidence for the interactions between Y5R and RhoA and their contribution to cytoskeleton remodeling and cell migration. We propose that the NPY/Y5R/RhoA axis is an essential pathway facilitating NB cell motility and their dissemination.

## Results

### Y5R Expression in NB Tissues and Cells Associates With a Migratory Phenotype

**Figure 1:**
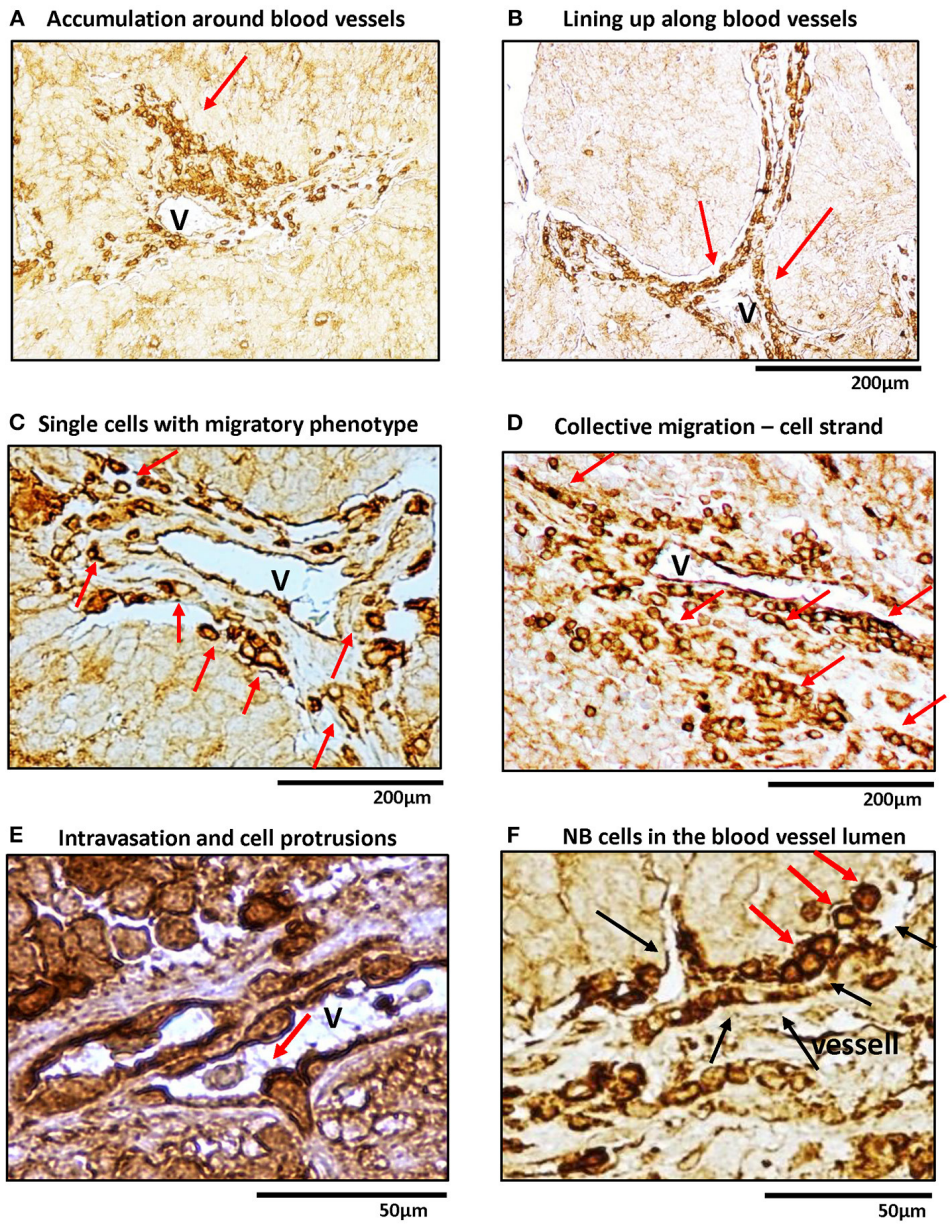
In human NB tissue, Y5R is preferentially expressed in cells with migratory and angioinvasive phenotypes. Immunohistochemistry with anti-Y5R antibody in undifferentiated and poorly differentiated human NB tissues. In all panels, red arrows indicate NB cells with the following phenotypes: **(A)** High Y5R expression in NB cells accumulated around a blood vessel. **(B)** Y5R-positive cells in alignment along blood vessels. **(C)** High Y5R expression in NB cells with a migratory phenotype consistent with single cell migration. **(D)** Groups of Y5R-positive NB cells with phenotypes consistent with collective migration. **(E)** An intravasating Y5R-positive NB cell with filopodia-like protrusions in the leading edge. **(F)** Y5R-positive cells in the blood vessel lumen. Black arrows indicate blood vessel walls.

Previously, we have shown that in human NB tissues, high Y5R expression is observed in a specific population of cells with an angioinvasive phenotype (Galli et al., 2016). To further investigate this phenomenon, we performed detailed histological analysis of human NBs immunostained for Y5R (Figure 1). Y5R-positive tumor cells aggregated around the blood vessels (Figure 1A) and lined up along the vessel walls (Figure 1B). Many of these Y5R-positive cells exhibited a morphology typical for single migratory or invasive cells, specifically manifested with their elongated and polarized shapes (Figure 1C). In addition, NB cells with strong Y5R immunoreactivity formed aligned groups, consistent with the strand mode of collective invasion (Figure 1D) (Lintz et al., 2017). Last, intravasating cells were also identified, with high Y5R expression at the leading edge, and some accumulation of the receptor at the trailing edge (Figure 1E). Importantly, the Y5R-positive leading edge of the intravasating cells formed long protrusions, which is characteristic for invading and migrating cells and consistent in morphology with filopodia (Figure 1E). In line with the above angioinvasive phenotype, highly Y5R-positive NB cells were also frequently observed in blood vessel lumens (Figure 1F). Altogether, the histological analysis of Y5R expression in NB tissues strongly suggested an association of Y5R expression with the invasive phenotype.

**Figure 2:**
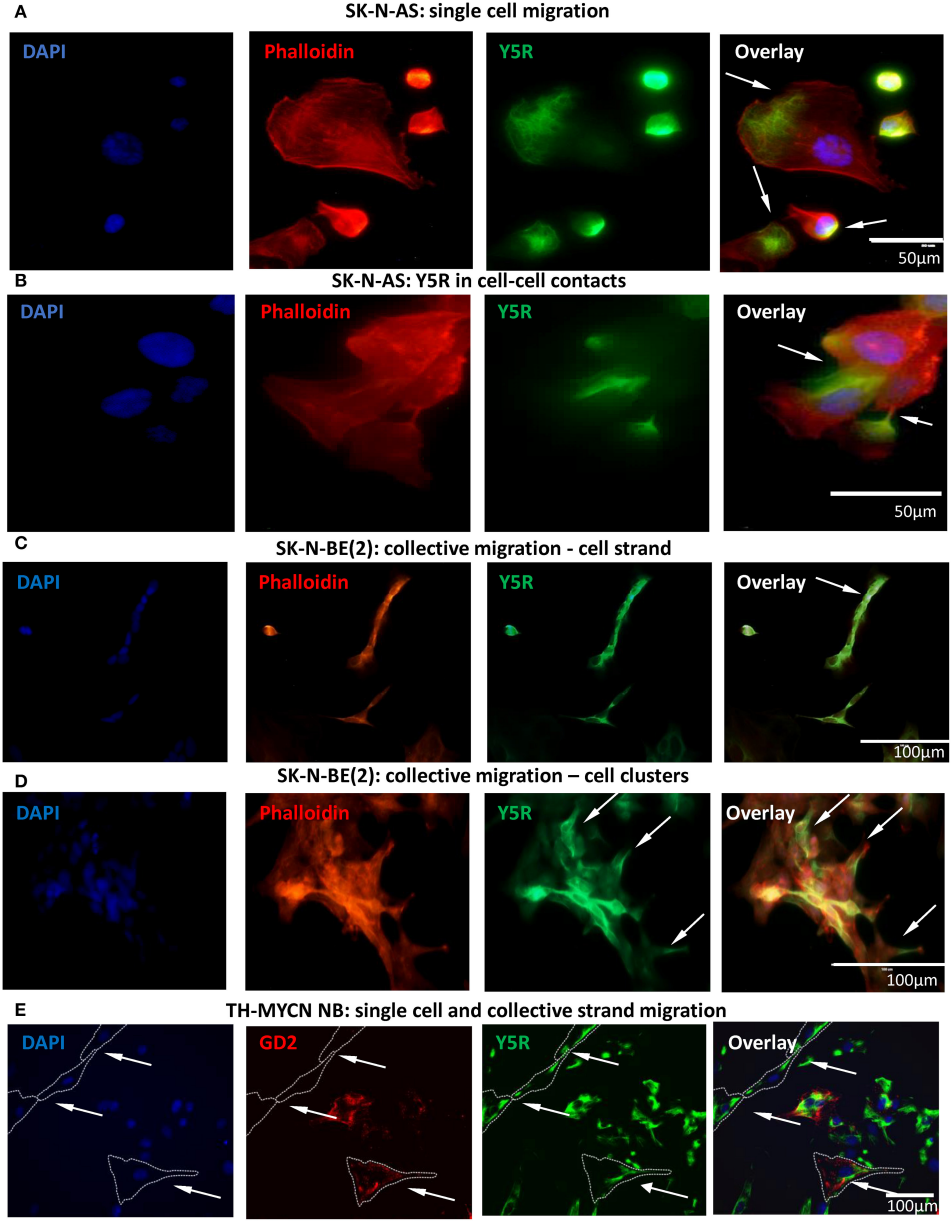
Y5R is expressed in defined areas of migratory NB cells. Immunocytochemistry with anti-Y5R antibody (green) in non-permeabilized NB cells detects Y5R on the cell surface **(A–D)**. The cells are counterstained with phalloidin (red) to label F-actin. In all panels, white arrows indicate Y5R expression in the following regions: **(A)** Y5R at the leading edges of SK-N-AS cells. **(B)** Y5R expression in SK-N-AS cell–cell junctions. **(C)** Y5R-positive SK-N-BE(2) cells aligned in a strand. **(D)** An SK-N-BE(2) cell cluster with high expression of Y5R in the leader cells. **(E)** Y5R expression in a single migratory cell and a migratory cell strand in a primary NB cell line from TH-MYCN mice. The cells were immunostained for Y5R (green) and an NB marker, GD2 (red).

To further investigate this phenomenon, we performed studies *in vitro*, using two human NB cell lines, SK-N-AS and SK-N-BE(2). To this end, non-permeabilized NB cells were immunostained for Y5R using an antibody that recognizes the extracellular N-terminus of the receptor. This approach allowed us to selectively detect the cellular distribution of the Y5R fraction present on the cell membrane and thereby able to bind the ligand. Subsequently, phalloidin was used to label F-actin and thereby assess cytoskeleton remodeling. In cells with a migratory phenotype, Y5R accumulated in specific regions of the cells, exhibiting the characterizing feature of polarized distribution on the cell edges. In SK-N-AS cells, the most profound Y5R expression was observed at the leading edge of single migratory cells, with a detectable co-localization between Y5R and phalloidin (Figure 2A). In addition, high Y5R expression was observed in cell–cell junctions (Figure 2B). The expression of Y5R in SK-N-BE(2) cells was more profound in groups of cells with morphology consistent with a collective migration phenotype. These included highly Y5R-positive cells aligned together and forming a chain characteristic for strand migration (Figure 2C). Moreover, in clusters formed by SK-N-BE(2) cells, more defined expression of Y5R was observed in cells with a migratory phenotype and those with morphology of leader cells that initiate cluster migration (Figure 2D). To confirm that the above phenomena are not artifacts associated with established cell lines, we used primary cultures of NB cells derived from tumors arising in TH-MYCN mice (Krawczyk et al., 2020). As in SK-N-AS and SK-N-BE(2) cell lines, in primary NB cells, Y5R accumulated in a polarized manner in edges of single migratory cells and those exhibiting the collective strand migration phenotype (Figure 2E). Hence, the subcellular distribution of Y5R in NB cells strongly implicated its role in the regulation of NB cell motility.

### NPY Stimulates Migration of NB Cells via Y5R and Y2R Interactions

**Figure 3:**
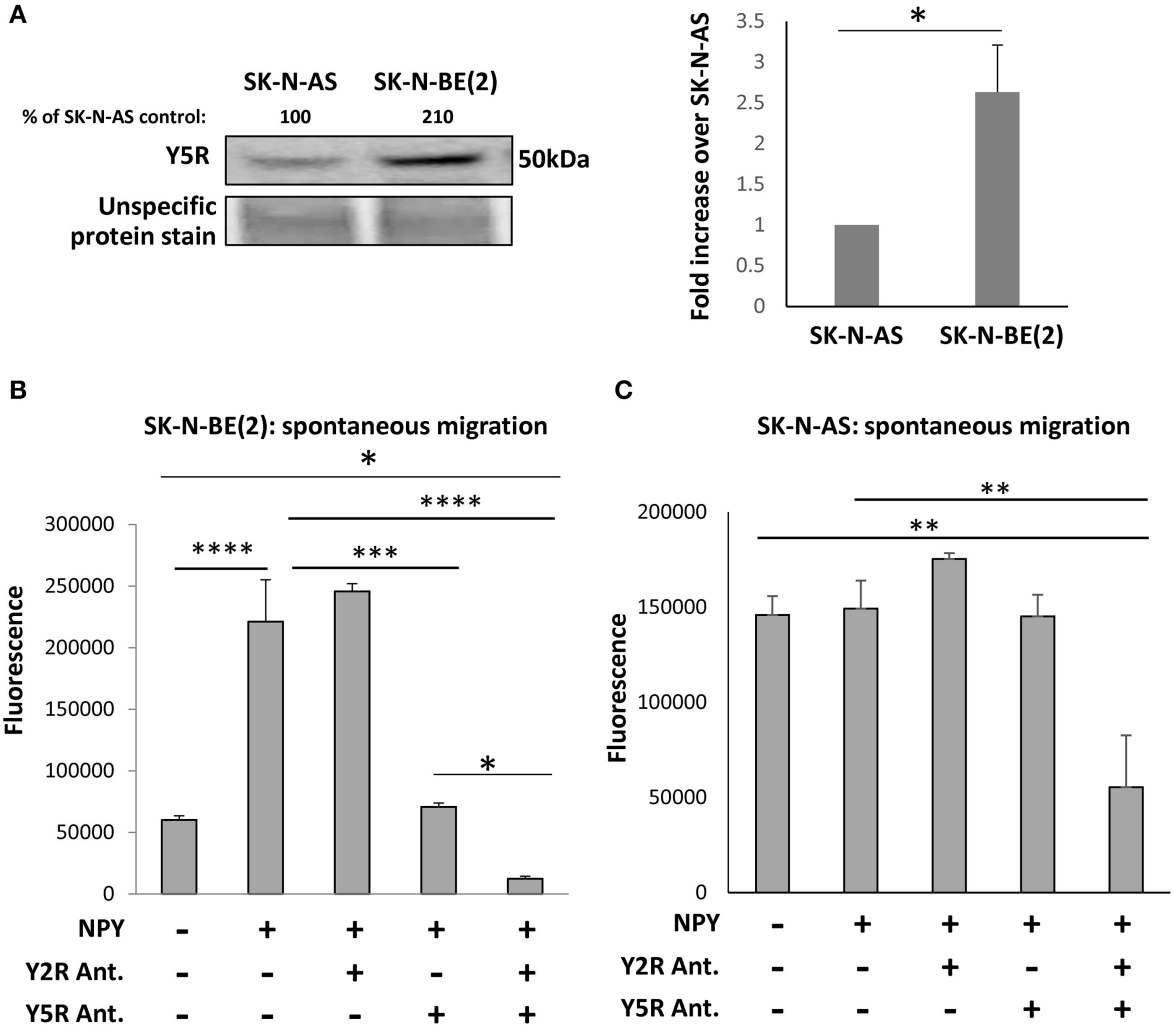
The NPY/Y5R axis promotes migration in NB cells. **(A)** Western blot analysis of Y5R expression in SK-N-AS and SK-N-BE(2) NB cell lines. *p < 0.05 by paired *t*-test, mean ± SEM, *n* = 4. **(B)** Spontaneous migration of SK-N-BE(2) cells treated for 22 h with NPY (10^−7^ M), in the presence or absence of Y2R antagonist (BIIE0246) and Y5R antagonist (CGP71683), each at a concentration of 10^−6^ M. **(C)** SK-N-AS cell migration in response to NPY (10^−7^ M), with or without Y2R and Y5R antagonists (10^−6^ M). **(B,C)** Spontaneous migration measured by a Transwell assay with NPY in both upper and lower chambers. *p < 0.05; **p < 0.01; ***p < 0.001; ****p < 0.0001 by one-way ANOVA followed by Tukey's test; mean ± SEM from two independent experiments, *n* = 4 each.

To evaluate the role of Y5R in cell motility, we used SK-N-AS and SK-N-BE(2) NB cell lines, which were derived from patients before therapy or after treatment with cyclophosphamide, respectively (Keshelava et al., 1998). As we have previously shown, chemotherapy induces Y5R expression in NB tissues and cell lines *in vitro* (Czarnecka et al., 2015). Indeed, Y5R protein levels were significantly higher in the SK-N-BE(2) cell line, compared to SK-N-AS cells (Figure 3A). These differences resulted in a differential response to NPY. The peptide increased spontaneous motility of SK-N-BE(2) cells, when applied to both the upper and lower chambers of the Transwell migration plate (Figure 3B). Y5R antagonist fully blocked the SK-N-BE(2) cell migration induced by exogenous NPY, while Y2R antagonist alone had no significant effect. Nevertheless, inhibition of both Y5R and Y2R further suppressed cellular migration below the baseline level (Figure 3B). On the contrary, treatment of SK-N-AS cells with NPY did not increase their migration (Figure 3C). However, the combined blocking of Y5R and Y2R resulted in a significant reduction of cell motility, compared to the control, suggesting a role for endogenous NPY in NB cell migration (Figure 3C). Moreover, the pattern of the response to NPY receptor inhibition suggested potential interactions between Y2R and Y5R.

### Overexpression of Y5R in CHO-K1 Cells Increases Their Motility

**Figure 4:**
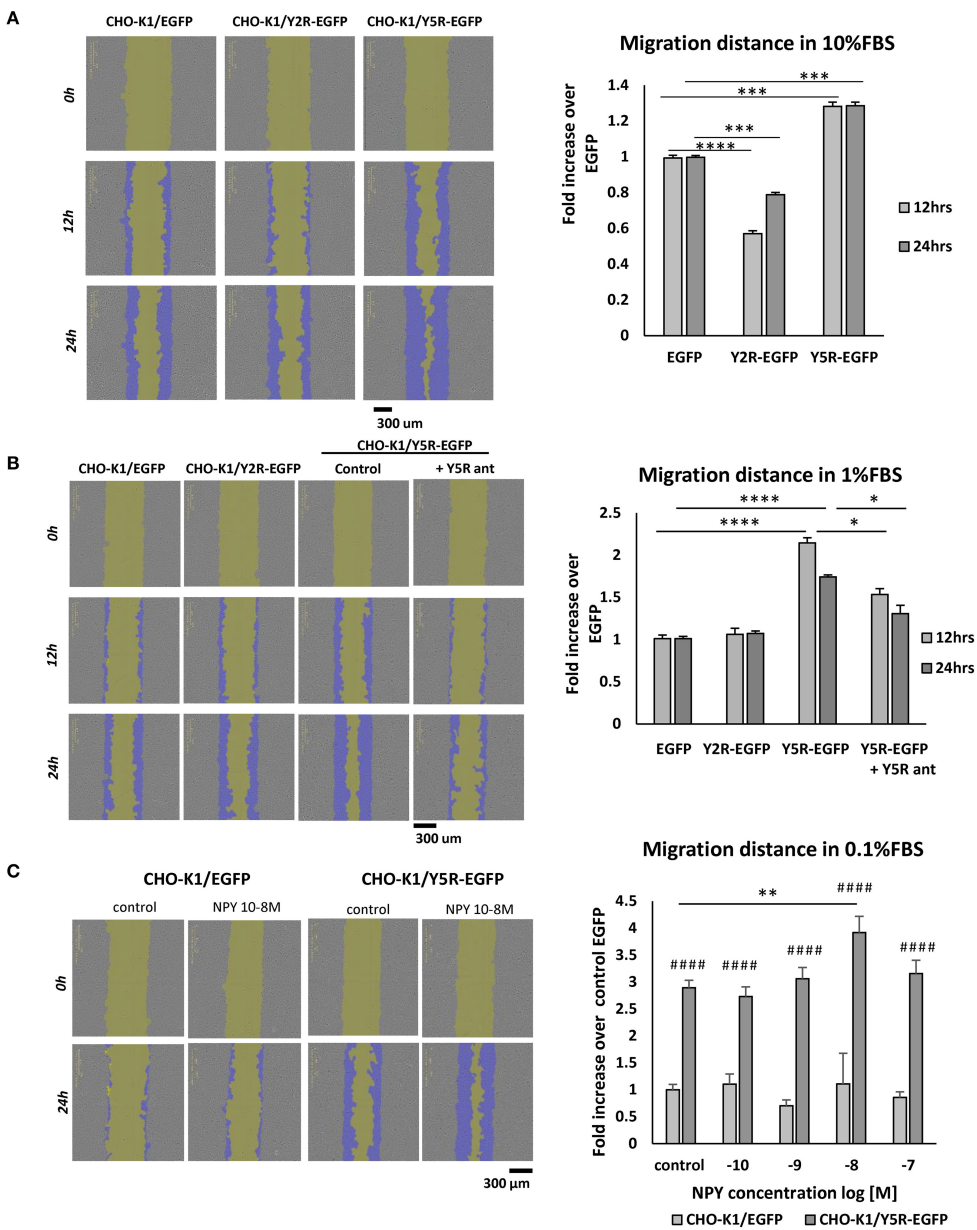
In CHO-K1 cells, overexpression of Y5R promotes cellular migration, while Y2R inhibits cell motility. Migration of CHO-K1 cells transfected with Y2R or Y5R fused to EGFP or with EGFP alone, measured by a wound healing assay with IncuCyte ZOOM live-cell imaging system analysis. Representative images captured by the IncuCyte software followed by quantitative analyses are shown. **(A)** Cell migration in 10% FBS. **(B)** Cell migration in 1% FBS. The migration of CHO-K1/Y5R-EGFP cells measured in the presence or absence of Y5R antagonist (CGP71683, 10^−6^ M). **(C)** Migration of CHO-K1/EGFP and CHO-K1/Y5R-EGFP cells in response to NPY (10^−10^-10^−7^ M) in 0.1% FBS. **(A–C)** *p < 0.05; **p < 0.01; ***p < 0.001; ****p < 0.0001 as shown or ^*####*^*p* < 0.0001 vs. CHO-K1/EGFP under the same conditions by one-way ANOVA followed by Tukey's test; mean ± SEM from three independent experiments, *n* = 3–32 each.

To dissect the role of particular NPY receptors in cellular migration, we used CHO-K1 cells transfected with Y2R or Y5R fused to EGFP (CHO-K1/Y2R-EGFP and CHO-K1/Y5R-EGFP cells, respectively), or with EGFP alone as a control (CHO-K1/EGFP) (Czarnecka et al., 2019). The motility of the above transfectants was compared using a scratch wound healing assay and the IncuCyte Live Cell Analysis System. The assay was performed at a high and low basal level of migration (10 and 1% FBS, respectively) to enable the detection of potential inhibitory and stimulatory effects of NPY receptor expression on cell migration. In 10% FBS, expression of Y5R increased CHO-K1 cell migration, compared to the CHO-K1/EGFP control, while Y2R expression exerted the opposite effect (Figure 4A). In 1% FBS, the stimulatory effect of Y5R on cell migration was more profound, while an inhibitory effect of Y2R was not detected (Figure 4B). Moreover, Y5R antagonist (CGP 71683) blocked the migratory effect of the receptor overexpression, confirming the specificity of the observed effects (Figure 4B). Stimulation with exogenous NPY further increased migration of CHO-K1/Y5R cells, although this effect was observed only at a concentration of 10^−8^ M (Figure 4C). Since NPY is present in FBS, the latter assay was performed in 0.1% FBS, to minimize the background activity of the peptide.

To control for potential interference of the effect of NPY receptors on cell proliferation with the assessment of cell motility, we compared proliferation levels of the tested cell lines using the IncuCyte Live Cell Analysis System, under conditions mimicking those employed in the migration assays. In 10% FBS, there were no significant differences in cell proliferation between cell lines, confirming that the observed changes in the rates of wound closure were due to the effects of NPY receptors on cell motility (Supplementary Figure 1A). In 1% FBS, proliferation of CHO-K1/Y5R cells was reduced, compared to other cell lines, further validating our migration data (Supplementary Figure 1B). Similarly, NPY had no stimulatory effect on CHO-K1/Y5R-EGFP cell proliferation in 0.1% FBS (Supplementary Figure 1C).

### NPY Acts as a Chemoattractant for Cells Expressing Y2R or Y5R

**Figure 5:**
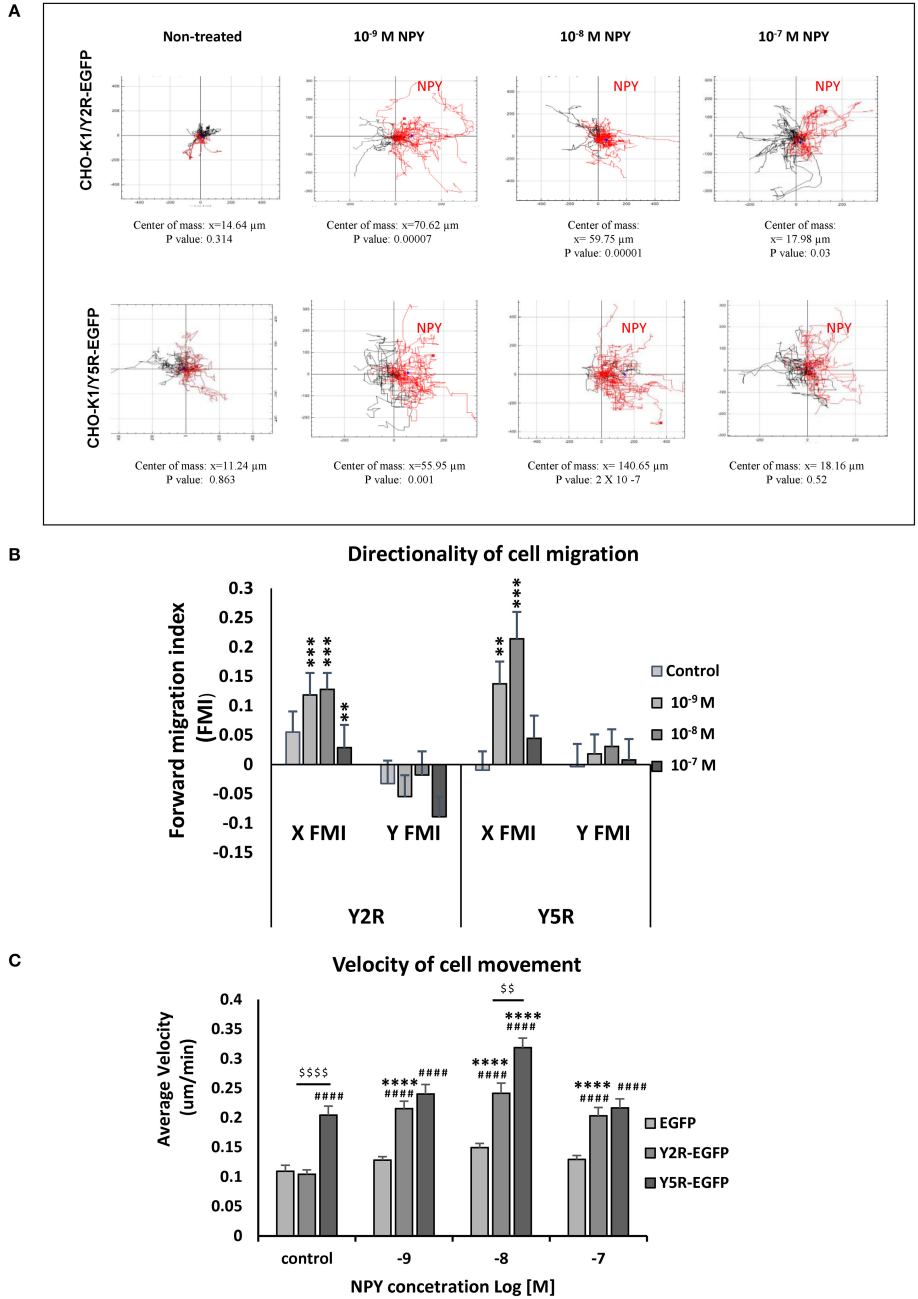
Y2R and Y5R mediate the chemotactic effect of NPY. **(A)** Trajectory plots showing tracks of individual CHO-K1/Y2R-EGFP or CHO-K1/Y5R-EGFP cells migrating in the chemotaxis chambers with various concentrations of NPY (10^−9^-10^−7^ M). Red tracks represent cells with net movement toward neuropeptide Y (NPY); black tracks represent cells migrating away from NPY. The center of mass represents the average of all single cell endpoints. *P*-values by the Rayleigh test for the uniformity of a circular distribution of points. **(B)** Quantification of the forward migration index (FMI) in a direction parallel (X) or perpendicular to the NPY gradient (Y). **p < 0.01; ***p < 0.001; X FMI vs. Y FMI for the given NPY concentration by *t*-test. **(C)** Average cell velocity for CHO-K1 transfectants under control conditions or upon treatment with NPY (10^−9^-10^−7^ M). ^####^*p* < 0.0001 vs. CHO-K1/EGFP control; ****p < 0.0001 vs. untreated control within the same cell line; ^$$$$^*p* < 0.0001 as shown, by one-way ANOVA followed by Tukey's test; mean ± SEM; *n* = 39–41 per condition.

Having established the role of NPY receptors in spontaneous cell migration, we sought to determine if NPY also acts as a chemotactic factor. To this end, CHO-K1 transfectants were plated in chemotaxis chambers with varying concentrations of NPY (10^−9^-10^−7^ M), and cells were tracked to compare the directionality of their movement (Figure 5A). Both Y2R and Y5R triggered chemotactic effects of NPY. In CHO-K1/Y2R-EGFP cells, an increase in the forward migration index toward NPY was observed at concentrations ranging from 10^−9^ to 10^−7^ M (Figure 5B). In CHO-K1/Y5R-EGFP cells, this effect was the most profound at an NPY concentration of 10^−8^ M (center of mass at the end of the experiment at 140.65 μm), while NPY at a concentration of 10^−7^ M had no chemotactic activity (Figures 5A,B).

In addition to the directionality of migration, an analysis of cell movement in the chemotaxis chamber revealed significant differences in migration velocity between the tested cell lines. In line with the results of the wound healing assay, CHO-K1/Y5R-EGFP transfectants had the highest velocity of movement under basal conditions (Figure 5C). The rate of their migration further increased upon stimulation with NPY at a concentration of 10^−8^ M. In contrast, cells transfected with Y2R-EGFP under basal conditions migrated at a rate comparable to the control CHO-K1/EGFP cells, while NPY increased the velocity of their movement at all concentrations tested (10^−9^-10^−7^ M) (Figure 5C).

### In Migratory Cells, Y5R Localizes in the Sites of Cytoskeleton Remodeling

**Figure 6:**
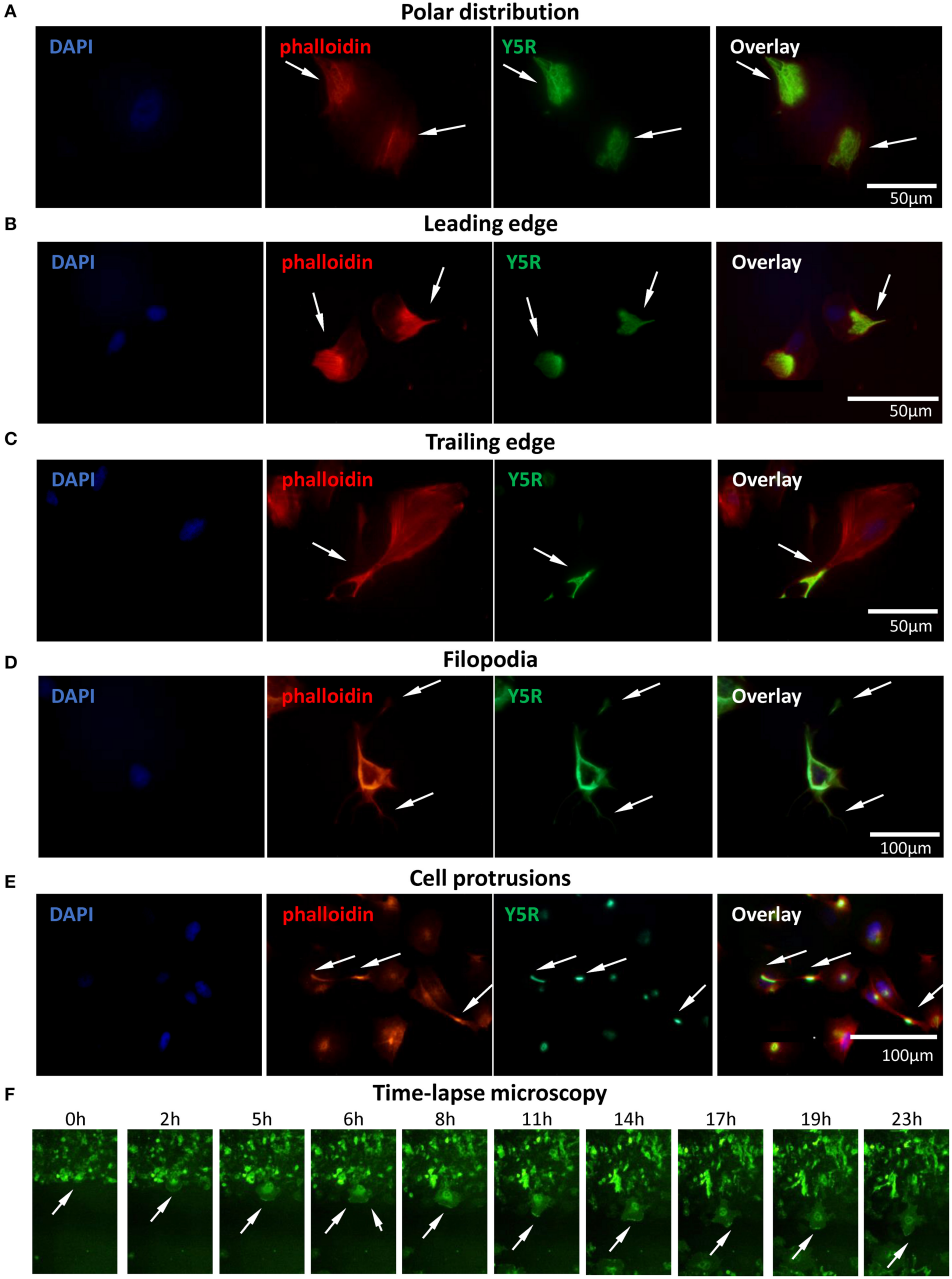
Surface Y5R is distributed in the areas of cytoskeleton remodeling in migratory CHO-K1/Y5R-EGFP cells. Immunocytochemistry with anti-Y5R antibody (green) in non-permeabilized CHO-K1/Y5R-EGFP cells counterstained with phalloidin (red) to detect F-actin. In all panels, white arrows indicate Y5R expression in the following regions: **(A)** Polar distribution of Y5R. **(B)** Y5R at leading edges of the migratory cells. **(C)** Y5R at the trailing edge of a migratory cell. **(D)** Y5R in filopodia and the areas of their formation. **(E)** Y5R in defined areas of the long CHO-K1/Y5R-EGFP cell protrusions. **(F)** Representative images of Y5R at the leading edges of the migrating CHO-K1/Y5R-EGFP cells via the IncuCyte ZOOM live-cell imaging system (0–23 h).

To further investigate the involvement of Y5R in cellular motility, we assessed its subcellular localization during cell migration. To this end, CHO-K1/Y5R-EGFP cells without prior permeabilization were immunostained with anti-Y5R antibody recognizing the extracellular domain of the receptor, as described above. In cells with a migratory phenotype, a polar distribution of the receptor was frequently observed (Figure 6A). Detailed analysis revealed the presence of Y5R at the leading and trailing edges of the migrating cells (Figures 6B,C). In addition, high Y5R immunoreactivity was observed in the cell membrane sections that developed multiple filopodia-like structures (Figure 6D). The sites with high Y5R expression were also observed in specific locations along the long cell protrusions (Figure 6E), consistent with the known localization of tip and shaft adhesion points described in filopodia (Jacquemet et al., 2015). To validate these findings and assess the dynamic changes of Y5R in migrating cells, we used time-lapse microscopy to monitor the movement and localization of Y5R receptors in CHO-K1/Y5R-EGFP cells during wound closure. As suggested by images captured in the fixed cells, Y5R underwent dynamic changes in localization during cell movement, with the predominant expression in lamellipodia-like protrusions present at the leading edge of the migrating CHO-K1/Y5R-EGFP cells (Figure 6F, Supplementary Movie 1). Hence, Y5R is localized to sites of crucial cytoskeleton remodeling that occurs during cell migration, suggesting a role for the NPY/Y5R axis in this process.

### NPY/Y5R Axis Promotes Filopodia Formation

**Figure 7:**
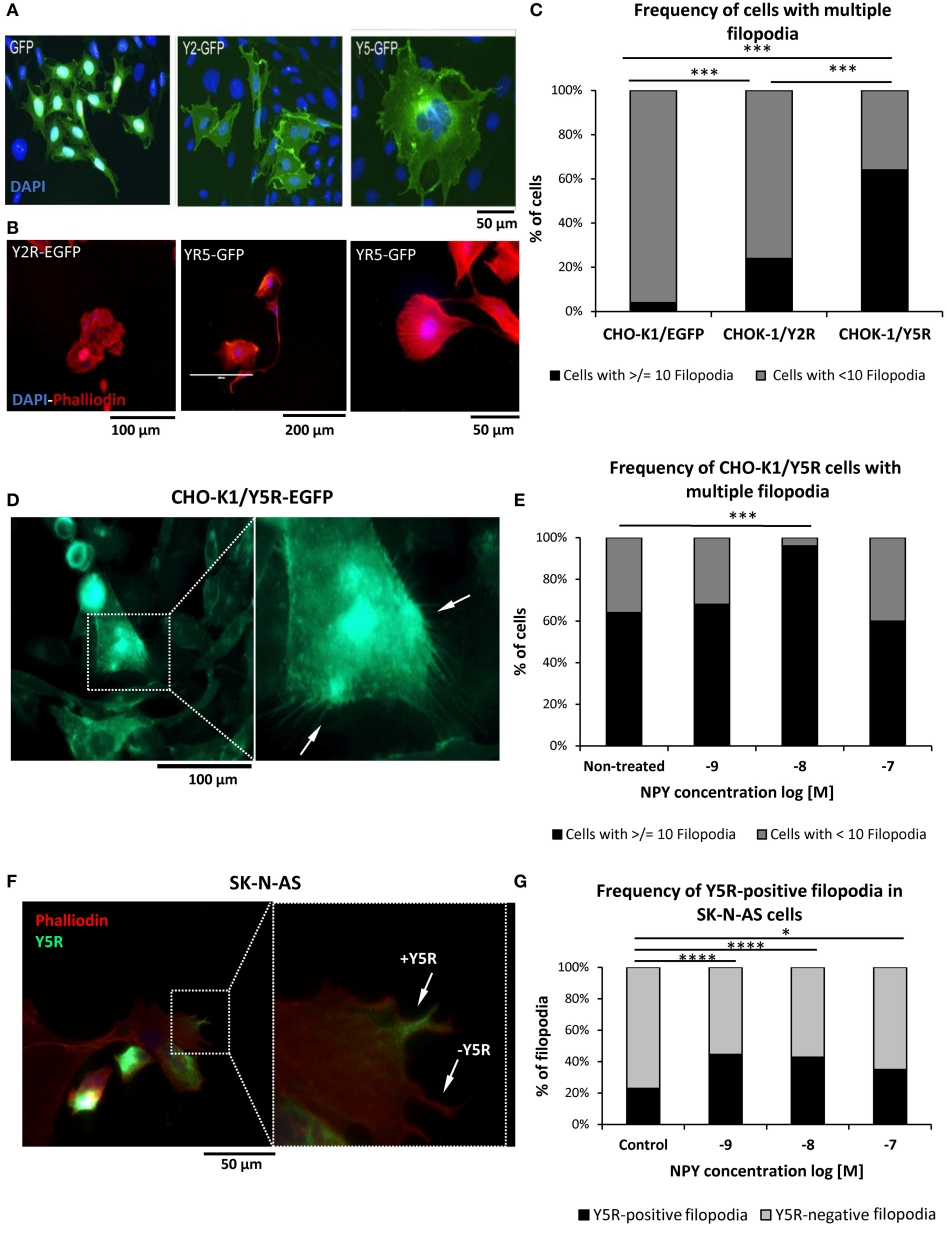
The NPY/Y5R axis contributes to filopodia formation. **(A)** Representative microscopic images of CHO-K1 cells transfected with EGFP alone or fused to Y2R or Y5R. **(B)** Representative images of CHO-K1/Y2R-EGFP and CHO-K1/Y5R-EGFP cells stained with Texas Red®-X phalloidin. **(C)** Frequency of cells with multiple filopodia (10 or more per cell) in CHO-K1/EGFP, CHO-K1/Y2R-EGFP, and CHO-K1/Y5R-EGFP cell lines. **(D)** A representative image of CHO-K1/Y5R-EGFP cells with multiple filopodia. **(E)** Effect of NPY on the frequency of CHO-K1/Y5R-EGFP cells with multiple filopodia. **(F)** A representative image of SK-N-AS NB cells with Y5R-postive and Y5R-negative filopodia stained with anti-Y5R antibody (green) and phalloidin (red). **(G)** Frequency of Y5R-positive filopodia in SK-N-AS cells with or without NPY (10^−9^-10^−7^ M). Panels **(C,E,G)**: *p < 0.05, ***p < 0.001; ****p < 0.0001 by Chi-square test; results shown as a percent of cells **(C,E)** or filopodia **(G)**; *n* = 25 per condition. CHO-K1/Y5R-EGFP and SK-N-AS shown and analyzed in **(C–G)** were cultured in wound healing assay chambers, and only cells at the migratory edges of wounds were analyzed.

Migrating cells heavily utilize filopodia, which are highly dynamic actin-rich membrane protrusions (Jacquemet et al., 2015). Hence, we compared their prevalence between CHO-K1 cells transfected with NPY receptors and EGFP alone. Cell morphology was compared based on EGFP fluorescence and phalloidin staining to evaluate the expression of NPY receptors and F-actin fibers, respectively (Figures 7A,B). Among all cell types tested, Y5R transfectants had the highest frequency of cells with multiple protrusions, including lamellipodia and filopodia-like structures (Figures 7A,B). Quantitative analysis revealed a slight increase in the prevalence of cells with multiple filopodia (10 or above per cell) in CHO-K1/Y2R-EGFP transfectants and a significantly higher frequency of such cells in the CHO-K1/Y5R-EGFP cell line (Figure 7C). Importantly, the areas of cell membrane particularly rich in Y5R also had high numbers of filopodia, which were positive for this receptor as well (Figure 7D). The number of CHO-K1/Y5R-EGFP cells with multiple filopodia was further increased upon treatment with NPY at a concentration of 10^−8^ M NPY (Figure 7E).

To confirm the relevance of our findings in CHO-K1 transfectants to NB, SK-N-AS cells growing at the edge of the scratch in the wound healing assay were immunostained for Y5R, followed by phalloidin staining to identify filopodia and assess expression of Y5R in these structures (Figure 7F). As seen in CHO-K1/Y5R-EGFP cells, NPY increased the number of Y5R-positive filopodia in SK-N-AS cells, with concentrations of 10^−8^ and 10^−9^ M being the most effective (Figure 7G). Altogether, these data provide evidence for a role for the NPY/Y5R pathway in filopodia formation and function.

### NPY/Y5R Axis Activates RhoA Pathway

**Figure 8:**
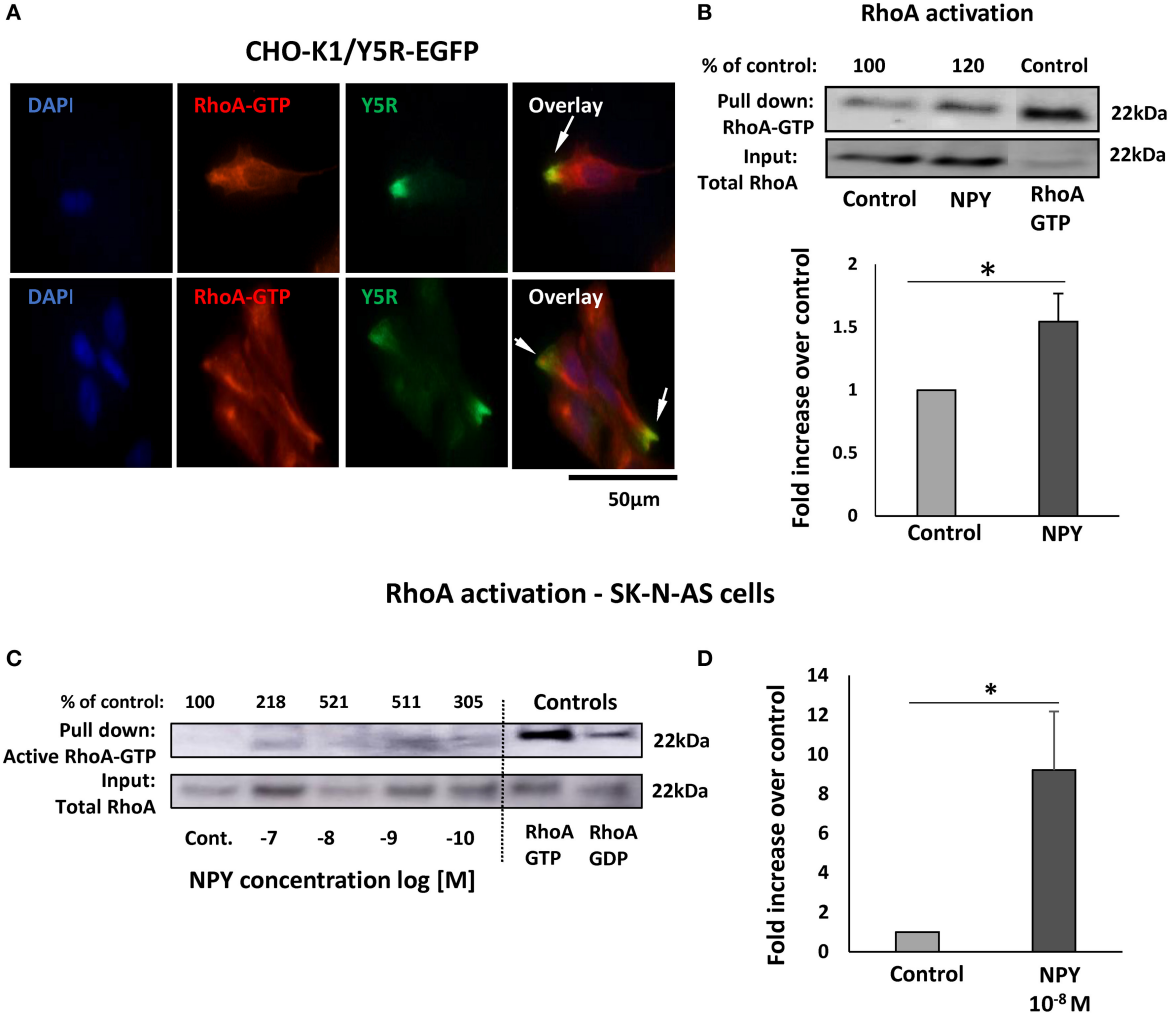
The NPY/Y5R pathway activates RhoA. **(A)** Immunocytochemistry with anti-Y5R (green) and anti-RhoA-GTP (red) antibodies in CHO-K1/Y5R-EGFP cells. **(B)** RhoA pull-down assay in CHO-K1/Y5R-EGFP cells treated with NPY (10^−7^ M). **(C)** RhoA pull-down assay in SK-N-AS NB cells treated with NPY (10^−10^-10^−7^ M). **(D)** Quantitative analysis of RhoA activity measured by a pull-down assay in SK-N-AS cells treated with NPY at a concentration of 10^−8^ M. **(C,D)** *p < 0.05 by *t*-test; mean ± SEM from three independent experiments.

Subcellular distribution of Y5R in migratory cells, including their leading and trailing edges, was consistent with commonly known sites of RhoA activation during cell migration (Ridley, 2015). Thus, we hypothesized that NPY/Y5R signaling promoted cellular migration through a RhoA-mediated mechanism. Indeed, in CHO-K1/Y5R-EGFP cells, Y5R present on the plasma membrane at the leading edges of cells with the migratory phenotype co-localized with an active RhoA-GTP (Figure 8A). This phenomenon was observed in single migratory cells and those in cell clusters. In agreement with this observation, RhoA pull-down assays revealed an increase in the level of active RhoA in CHO-K1/Y5R cells upon stimulation with NPY (Figure 8B).

To confirm that NPY/Y5R/RhoA signaling pathway is active in NB, a RhoA pull-down assay was performed in SK-N-AS cells. The initial dose-response experiment indicated NPY-induced RhoA activation, with peaks of activity at NPY concentrations of 10^−8^ and 10^−9^ M (Figure 8C). Hence, in subsequent RhoA pull-down assays, we have focused on the NPY concentration of 10^−8^ M, as the most effective in the previous experiments, and found a significant increase in RhoA activity in the NPY-treated SK-N-AS cells (Figure 8D).

**Figure 9:**
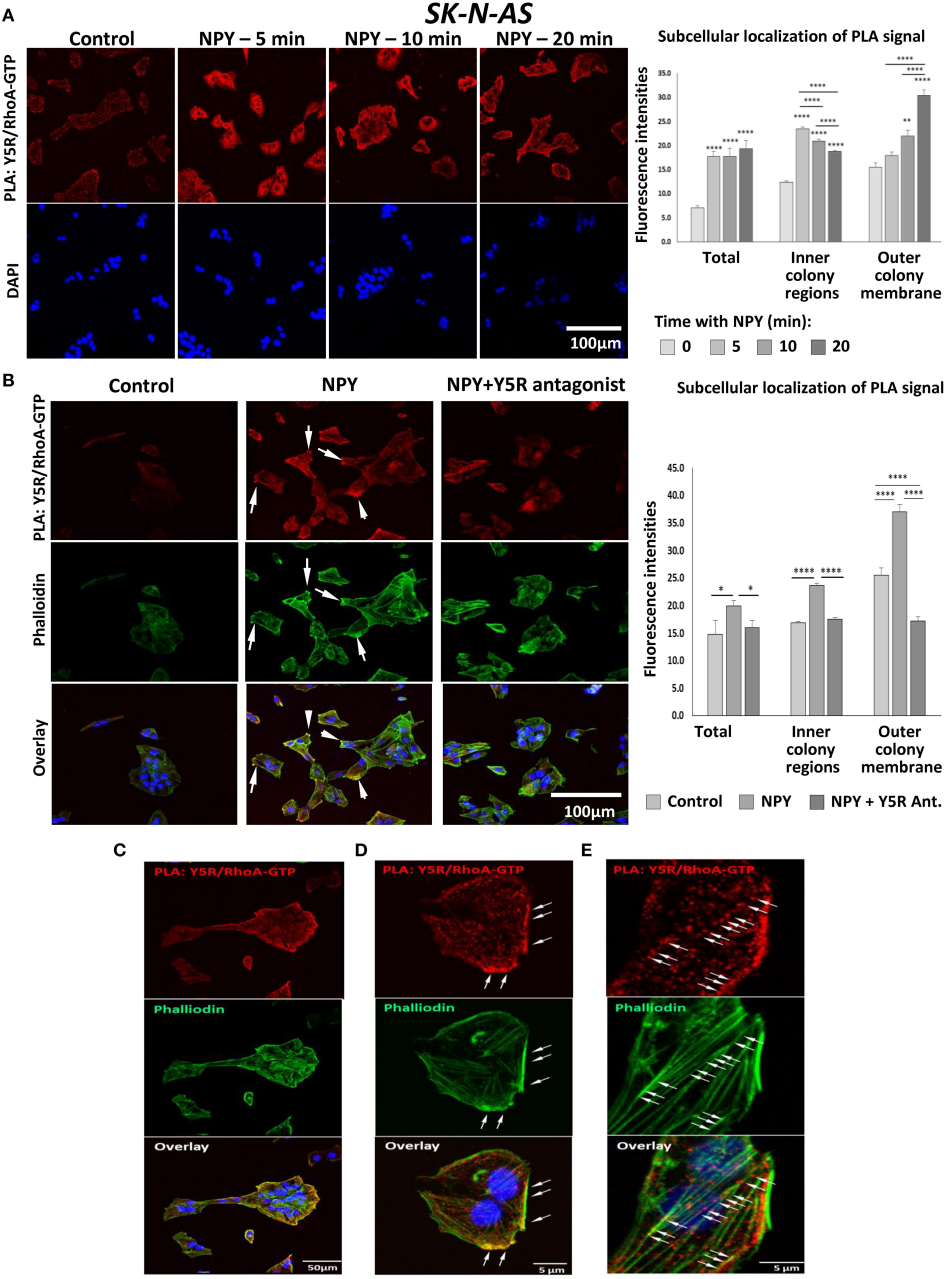
*In situ* proximity ligation assay (PLA) reveals interactions between Y5R and RhoA-GTP. PLA performed in SK-N-AS cells with anti-Y5R and anti-RhoA-GTP antibodies. **(A)** Representative confocal microscopy images of PLA in SK-N-AS cells treated with NPY (10^−8^ M) for 5, 10, and 20 min. Red dots represent interactions between Y5R and RhoA-GTP. The graph depicts quantification of fluorescence intensities for the total PLA signal or the signal located inside of the cell colonies and near their outer membranes in SK-N-AS cells treated with NPY. **(B)** PLA (red) and phalloidin staining (green) in SK-N-AS cells treated with NPY (10^−8^ M) for 20 min, with or without Y5R antagonist (10^−6^ M) pre-treatment for 30 min. White arrows indicate leading edges of the neuroblastoma (NB) cells. The graph represents a quantification of fluorescence intensity of the PLA signal—total or located inside of cell colonies and near their outer membranes in SK-N-AS cells treated with NPY and Y5R antagonist as above. **(A,B)** *p < 0.05, **p < 0.01, ****p < 0.0001 vs. non-treated control or ****p < 0.0001 as indicated, by one-way ANOVA followed by Tukey's test; mean ± SEM, *n* = 11–21 per condition. **(C)** Co-localization of the PLA signal (red) with F-actin (green) on the outer membranes of cells within the colony with migratory phenotype. **(D)** PLA signal in the area rich in F-actin near the outer plasma membrane. **(E)** Localization of the PLA signal along F-actin fibers.

To determine whether NPY-induced RhoA activation was mediated by Y5R, a proximity ligation assay (PLA) with anti-Y5R and anti-RhoA-GFP antibodies was employed to assess their direct interactions. Treatment with NPY at a concentration of 10^−8^ M, which was the most effective in stimulating cell migration and cytoskeleton remodeling, significantly increased the intensity of the PLA signal in SK-N-AS cells, starting at 5 min after NPY administration (Figure 9A). Initial Y5R-RhoA-GTP interactions observed 5 min after stimulation were located intracellularly, in the perinuclear areas of the cells. However, 20 min after NPY administration, the intensity of the cytosolic signal decreased, while the Y5R-RhoA-GTP interactions occurred mainly on the outer membranes of cells present at the edges of cell colonies (Figure 9A). The subsequent co-staining with phalloidin revealed a localization of the PLA signal in areas rich in F-actin, particularly in the protrusions of cells with a morphology consistent with that of leader cells during collective migration (Figure 9B). No increase in Y5R-RhoA-GTP interactions was observed when NPY was applied in the presence of Y5R antagonist, confirming the specificity of the PLA signal (Figure 9B). The membranous localization of the interaction events between Y5R and RhoA-GTP was particularly apparent in single cells with migratory phenotypes and outer cells in the colonies with a morphology consistent with collective migration (Figure 9C). High magnification images confirmed a strong co-localization of the PLA signal with F-actin fibers along the plasma membrane (Figure 9D). However, PLA signals located away from the plasma membrane were also distributed along F-actin fibers (Figure 9E). Altogether, these findings supported a role for the NPY/Y5R pathway in RhoA activation and the subsequent cytoskeleton remodeling involved in cell migration.

### NPY in Cell Invasion

**Figure 10:**
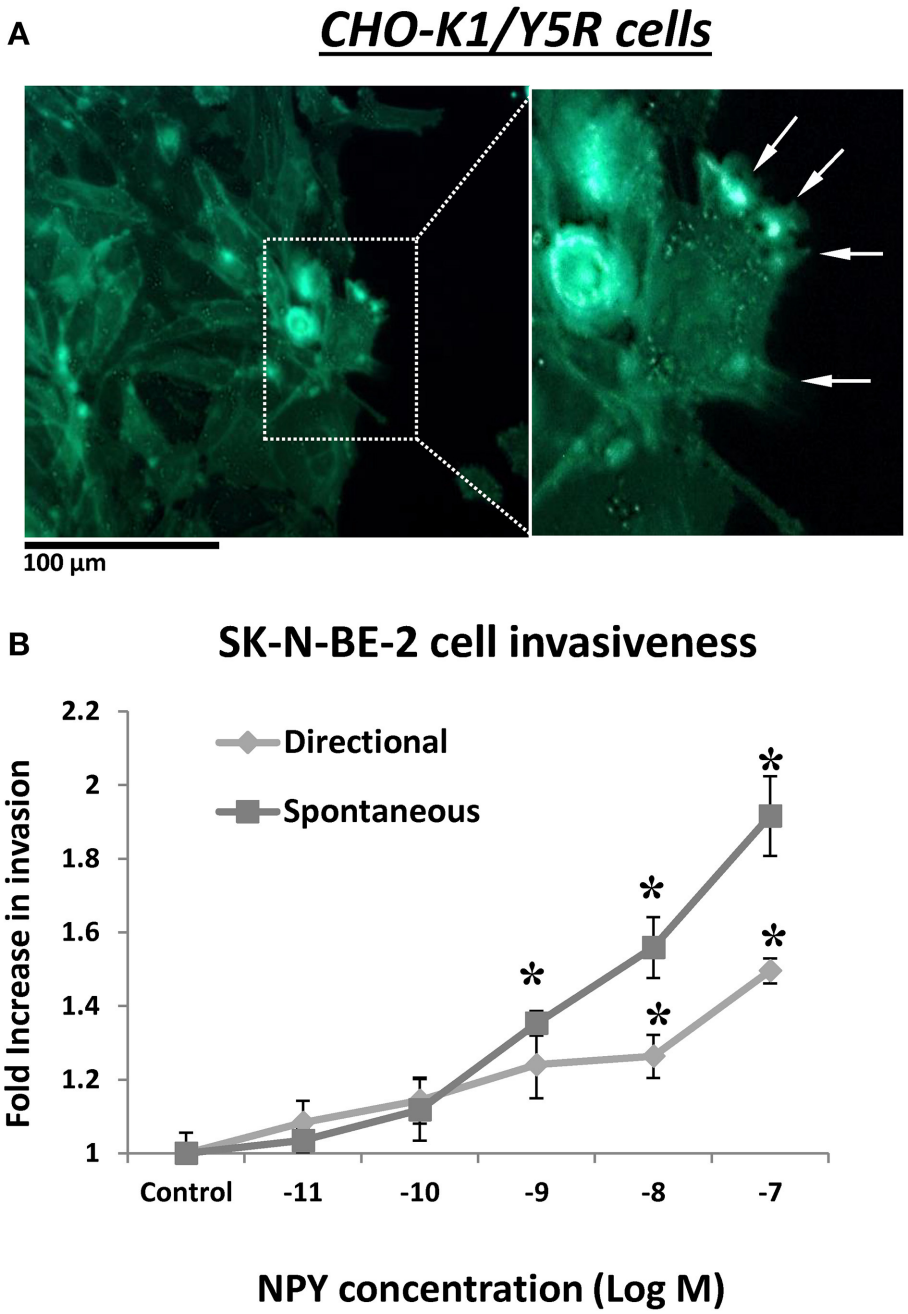
NPY/Y5R axis is involved in cell invasiveness. **(A)** Representative microscopic image of CHO-K1/Y5R cells invading Matrigel. White arrows indicate invadopodia. **(B)** NPY-induced SK-N-BE(2) cell invasiveness measured in a Matrigel-coated Transwell plate. The cells were subjected to NPY (10^−11^-10^−7^ M) for 22 h in the lower chamber of the Transwell plate (directional invasiveness) or both upper and lower chambers (spontaneous invasiveness). *p < 0.05 by one-way ANOVA followed by Dunnett's test; mean ± SEM, *n* = 4.

In addition to its role in cell migration, our data suggested a role for the NPY/Y5R pathway in cell invasiveness as well. In CHO-K1/Y5R-EGFP cells subjected to a wound healing assay on Matrigel-coated plates, Y5R was present in protrusions at the leading edge of the invading cells, with a morphology consistent with invadopodia and filopodia (Figure 10A). Furthermore, NPY stimulated invasiveness of SK-N-BE(2) NB cells in Matrigel-coated Transwell plates (Figure 10B). In contrast to the migration assay, here NPY exerted a dose-dependent effect, with the highest stimulation observed at a concentration of 10^−7^ M. The peptide stimulated both spontaneous invasiveness when no NPY gradient was present and directional cell invasion when it served as a chemotactic agent (Figure 10B).

## Discussion

Previous data indicated a role for the NPY system in several aspects of NB biology (Tilan and Kitlinska, 2016). However, the role of the peptide in its dissemination has not been tested directly. Here, we have demonstrated for the first time that NPY, both tumor-derived and exogenous, stimulates NB cell migration. We have also identified Y5R as the main NPY receptor mediating its effect on cell motility and elucidated the mechanisms of its actions.

In NB tissues at diagnosis, Y5R was preferentially expressed in tumor cells with angioinvasive phenotypes. Notably, in both tumor tissues and cell culture, we observed strands of highly Y5R-positive NB cells. This phenotype is consistent with the strand mode of migration typical for neural crest cells during development (Druckenbrod and Epstein, 2005). Hence, the Y5R-rich invasive fraction of NB cells may represent a more primitive cell sub-population, perhaps with the cancer stem cell phenotype previously shown to be responsible for NB dissemination and chemoresistance (Bahmad et al., 2019). In line with these morphological features of Y5R-positive cells, NPY stimulated NB cell migration *in vitro* via Y5R. These findings agree with previous reports of the role for Y5R in cancer cell motility. In another NPY-rich tumor, Ewing sarcoma, Y5R was involved in the migration of hypoxic cancer stem cells (Tilan et al., 2013). The same NPY/Y5R pathway stimulated motility of breast and hepatic cancer cells, while high Y5R expression was detected at the invasive edge in liver cancer tissue (Medeiros et al., 2011; Dietrich et al., 2020). Hence, the NPY/Y5R axis contributes to the migratory phenotype in a variety of malignancies, implicating its activity as a pan-cancer mechanism underlying tumor cell dissemination. In support of this, we were able to trigger a migratory phenotype and enhance the motility in CHO-K1 cells by inducing Y5R expression.

Despite evidence for the role of NPY in stimulating cell motility and invasiveness, the exact mechanisms underlying its actions remained unknown. Here, we have shown for the first time a role for the NPY/Y5R pathway in the regulation of RhoA activity and subsequent cytoskeleton remodeling involved in cell movement. As GPCRs, NPY receptors act via the Gα_i_ subunits (Balasubramaniam, 1997). Hence, the Y5R-induced RhoA activation was an unexpected finding, since interactions between GPCRs and RhoA pathway are typically attributed to the actions of Gα_12/13_ and Gα_q_ subunits (Yu and Brown, 2015). Nevertheless, there are reports of Gα_i_-induced RhoA activation in peripheral blood leukocytes, which is mediated by PI3K (Huang et al., 2001). Thus, further studies are required to determine the exact signaling events leading to Y5R-induced RhoA activation.

In line with its stimulatory effect on RhoA, sub-cellular localization of the Y5R fraction that was located on the cell surface, and therefore able to bind its ligand, was consistent with areas of high RhoA activity during cell migration (Ridley, 2015). This included the leading and trailing edges of the single migratory cells, as well as cell membrane areas with high filopodia content. In cell clusters, a high surface Y5R expression was seen in leader cells and NB cells migrating as strands. Of particular interest was a localization of the PLA signal on the outer edges of NB cell colonies, where RhoA drives the formation of actomyosin cables preserving the integrity of the migrating cell units (Zegers and Friedl, 2014). Notably, the initial Y5R/RhoA-GTP interactions were observed in all cells in the cluster, followed by its accumulation in the outer cell membranes. Further studies are needed to elucidate the mechanisms directing Y5R/RhoA activation to these specific locations. Last, high Y5R expression was seen at the cell-cell junctions, which are crucial for collective migration and have been shown to be RhoA dependent (Zegers and Friedl, 2014). Importantly, NPY/Y5R axis facilitated the formation of filopodia, which initiate the earliest cellular contacts and subsequent formation of such junctions (Ridley, 2015). Hence, NPY/Y5R activation appears to contribute to multiple functions that RhoA plays in cytoskeleton remodeling during single cell and collective migration. As similar processes were observed in NB cells and CHO-K1/Y5R transfectants, the activation of the Y5R/RhoA axis may be a common mechanism underlying NPY pro-migratory activity in various cells.

Aside from its effect on spontaneous cell motility, Y5R also mediated a chemotactic effect of NPY. This Y5R-dependent chemotaxis may be associated with the presence of these receptors in filopodia, which participate in environment sensing (Jacquemet et al., 2015). Subsequent directional activation of the membrane receptors present in filopodia is essential for directional cell movement (Ridley, 2015). In both NB and CHO-K1/Y5R cells, Y5R was located uniformly along filopodia. However, in long filopodia-like protrusions, Y5Rs were present in specific areas along these structures, suggesting its localization to adhesion points involved in extracellular matrix sensing or Y5R transport to the filopodia tips (Jacquemet et al., 2015). Further studies are required to fully elucidate functions of Y5R in these specialized cell structures.

One of the characteristic features of the effects of the NPY/Y5R pathway on processes associated with cellular motility, such as spontaneous migration, chemotaxis, and filopodia formation, was the lack of a dose-dependent increase in their intensity. For all of these processes, the peak of NPY activity was observed at a concentration of 10^−8^ M, while the lower and higher concentrations were less effective or had no effect. This selective, concentration-dependent activity of the NPY/Y5R axis may be associated with tight regulation of RhoA during cell migration. To enable coordinated cytoskeleton remodeling in various sub-cellular compartments of migrating cells, RhoA activity is regulated spatially and temporally (Ridley, 2015; Bolado-Carrancio et al., 2020). While RhoA is necessary for cell movement and its insufficient activation impairs this process, the over-activation of RhoA signaling leads to stress fiber formation and inhibition of cell migration (Ridley, 2015). Thus, the NPY concentration of 10^−8^ M may provide an optimal level of RhoA activation for cell motility. This phenomenon may facilitate intravasation of cancer cells in NPY-rich tumors, such as NB. As these tumors secrete high levels of NPY, it is plausible that local concentrations of the peptide are higher in the tissues than in the blood stream. Hence, the Y5R-positive cells may be prone to intravasation into the vessel lumen containing a lower NPY concentration. This notion is supported by a characteristic distribution of Y5R-positive cells around the blood vessels and their frequent intravasation. Interestingly, the effect of NPY on NB cell invasiveness exhibited a linear dose dependence, suggesting the involvement of additional, potentially RhoA-independent mechanisms in interactions with extracellular matrix and its degradation.

Although our studies clearly identified Y5R as the main NPY receptor mediating its pro-migratory effects, NPY actions can be enhanced by the interactions between its heterotypic receptors. This may involve a heterodimer formation between Y1R and Y5R, as well as indirect interactions between Y2R and Y5R (Gehlert et al., 2007; Kilpatrick et al., 2015; Czarnecka et al., 2019). In the latter case, despite the lack of evidence for Y2R and Y5R dimerization, the ligand binding to one of the receptors triggers transactivation of the other receptor and subsequent signaling cross-talk (Czarnecka et al., 2019). Hence, inhibition of both receptors is required to prevent NPY actions in this setting. Both direct and indirect NPY receptor interactions enable the response to NPY at concentrations significantly below their known affinities, which is not seen in cells expressing one type of NPY receptors (Movafagh et al., 2006; Pons et al., 2008; Czarnecka et al., 2019). Although this phenomenon was most extensively characterized with respect to the mitogenic effects of NPY, similar co-activation of heterotypic NPY receptors has been shown to increase its migratory effect in endothelial cells (Movafagh et al., 2006; Pons et al., 2008; Czarnecka et al., 2019). Consistent with this, our previous studies indicated that the NPY-induced migration of hypoxic Ewing sarcoma cells with a cancer stem cell phenotype requires Y2R and Y5R interactions (Tilan et al., 2013). NB cells constitutively express both NPY and Y2R (Kitlinska et al., 2005; Lu et al., 2010). This autocrine NPY/Y2R loop maintains NB cell proliferation (Kitlinska et al., 2005; Lu et al., 2010). Here, we have shown that Y2R cooperates with Y5R in stimulating NB cell migration as well. While in Y5R-rich SK-N-BE(2) cells the Y5R antagonist fully blocked the migratory effects of exogenous NPY, combined Y2R and Y5R antagonists were required in both SK-N-AS and SK-N-BE(2) cell lines to inhibit the basal level of cell motility driven by the endogenous peptide. Hence, Y2R/Y5R interactions are necessary to enable NB cell migration at low NPY levels, while elevated Y5R expression facilitates the response to high NPY concentrations.

To further dissect the role of the particular NPY receptors in cell migration, we used CHO-K1 cells, which express negligible basal levels of NPY receptors and transfected them with Y2R or Y5R (Czarnecka et al., 2019). As expected, CHO-K1/Y5R cells had a migratory phenotype, rich in lamellipodia and filopodia, increased spontaneous motility, and directional migration toward NPY. Surprisingly, however, expression of Y2R alone decreased motility of CHO-K1 cells. Nevertheless, NPY exerted a chemotactic effect in CHO-K1/Y2R transfectants, which was associated with a slight increase in the number of filopodia observed in this cell line. Hence, the net effect of NPY on cell motility and directional migration depends on the interplay between its heterotypic receptors. It is possible that at high NPY concentrations, the presence of Y2R modulates Y5R-dependent RhoA activation, preventing its overactivation and subsequent inhibition of cell motility (Ridley, 2015). In contrast, Y2R may enhance the effects of Y5R via indirect interactions when the ligand availability is low. Importantly, Y5R has also been shown to interact with other membrane receptors, such as a neurotrophin receptor, TrkB, which facilitates its transactivation by brain-derived neurotrophic factor (BDNF) (Czarnecka et al., 2015). Further research is required to determine the exact mechanisms of these interactions and their effects on NPY actions.

In summary, our results provided evidence for the role of NPY in NB cell migration and identified Y5R/RhoA-mediated effects on cytoskeleton remodeling as the mechanism of its actions. Our findings provide a scientific foundation for future investigations into the role of the NPY/Y5R axis in NB metastasis using relevant animal models. Importantly, while Y5R expression in non-treated NB tumors is limited to the sub-population of the angioinvasive cells, the levels of Y5R and NPY increase in cells exposed to cytotoxic treatment (Czarnecka et al., 2015). As we have previously shown, this inducible NPY/Y5R autocrine loop promotes cell survival under pro-apoptotic conditions and contributes to chemoresistance (Czarnecka et al., 2015). Consequently, in post-treatment NB tumors, all surviving NB cells are highly Y5R-positive (Czarnecka et al., 2015). Hence, given the role of the NPY/Y5R axis in cell motility, this pathway may be particularly important in secondary dissemination of treatment-resistant NBs. Altogether, the data presented here, along with our previous reports indicating the inhibitory effect of NPY receptor blockage on primary tumor growth via anti-proliferative and anti-angiogenic activity of the Y2R antagonist or pro-apoptotic actions of the Y5R antagonist, warrant pre-clinical investigations aiming at an assessment of Y2R and Y5R as therapeutic targets for NB (Lu et al., 2010; Czarnecka et al., 2015). Such therapies may be particularly relevant to recurrent and refractory tumors, which thus far lack adequate treatment. Moreover, as Y5R and NPY expression have been shown in other metastatic tumors, blocking this pathway may become an effective anti-metastatic therapy for malignancies other than NB (Kitlinska et al., 2005; Medeiros et al., 2011; Tilan et al., 2013; Ueda et al., 2013; Tjon-Kon-Fat et al., 2018; Dietrich et al., 2020).

## Materials and Methods

### Reagents

NPY was purchased from Bachem (San Carlos, CA). Y5R antagonist, CGP71683, and Y2R antagonist, BIIE0246, were obtained from Tocris (Ellisville, MO).

### Human Tissue Sample Analysis

Tissue sections from 87 pediatric patients with neuroblastic tumors at diagnosis, collected between the years 2004 and 2009, were obtained from Children's Oncology Group (COG). These samples were collected by COG institutions upon obtaining appropriate consents, and their use was approved by the Georgetown University Institutional Review Board. Immunohistochemistry on the above samples was performed using rabbit polyclonal anti-Y5R antibody (1:300) (Novus Biologicals, Littleton, CO, cat # NBP1-00957).

### Cell Culture

Human NB cell lines, SK-N-AS and SK-N-BE(2), were obtained from American Type Culture Collection (ATCC, Manassas, VA) and cultured in DMEM media or EMEM:F12K (1:1) media with 10% FBS, respectively. CHO-K1 cells were obtained from ATCC and cultured in F12K media supplemented with 10% FBS. Stable transfectants expressing NPY receptors fused to EGFP or EGFP alone were developed as previously described (Czarnecka et al., 2019). The NB cell lines from tumors developing in TH-MYCN mice were isolated using conditional reprogramming technology and cultured under 2% oxygen in the F medium containing DMEM + F12 nutrient mix (3:1 v/v) supplemented with 10 μM ROCK inhibitor, Y-27632, 5 μg/ml insulin (Sigma-Aldrich, St. Louis, MO), 0.1 nM cholera toxin (Sigma-Aldrich), 0.125 ng/ml epidermal growth factor (EGF) (Life Technologies, Carlsbad, CA) and 25 ng/ml hydrocortisone (Sigma-Aldrich), as previously described (Krawczyk et al., 2020).

### Western Blot

Cell membrane proteins were isolated from SK-N-AS and SK-N-BE(2) cells, as previously described (Pfeiffer et al., 2001). Upon SDS-PAGE and protein transfer, the membranes were stained using Pierce™ Reversible Protein Stain Kit for Nitrocellulose Membrane (Thermo Fisher Scientific, Waltham, MA). The density of the total unspecific protein staining per each well was measured using Image J software and used as a loading control. Western blot was performed using goat polyclonal anti-Y5R antibody (Everest Biotech, Ramona, CA, cat # EB06769). Densitometry was performed as above and the band intensities were normalized to the unspecific protein stain.

### Transwell Migration and Invasion Assay

The BD FluoroBlok™ 96-well Transwell plate or BD BioCoat FluoroBlok™ tumor invasion systems (BD Biosciences, San Jose, CA) were used to evaluate NB cell migration and invasion, respectively. NB cells, SK-N-AS, and SK-N-BE(2), were suspended in their respective media supplemented with 5% FBS and seeded in the upper chambers at a density of 2.5 × 10^4^ cells per well. The effect of NPY on spontaneous migration was tested by adding the same concentrations of the peptide, with or without Y2R and Y5R antagonists, to both upper and lower chambers of the plate. The Transwell migration plate was then incubated for 22 h at 37°C, in 5% CO_2_, followed by staining with calcein AM at a concentration of 4 μg/ml in Hank's Balanced Salt Solution (HBSS, Thermo Fisher Scientific). The fluorescence was measured from the bottom of the migration plates using EnSpire Multimode Plate Reader (Perkin Elmer, Waltham, MA).

### Scratch Wound Healing Assay Using IncuCyte Live Cell Imaging System

CHO-K1 cells transfected with EGFP, Y2R-EGFP, and Y5R-EGFP were seeded in IncuCyte® ImageLock 96-well plates at a density of 2–2.5 × 10^5^ cells per well. Eighteen hours after seeding, a scratch was made in the confluent monolayer using the 96-well Wound Maker™. Cells were then washed with serum-free medium to clear any floating cells within the scratch prior to treatment. The subsequent migration monitoring was performed in media with three different serum concentrations, 10%, 1%, or 0.1% FBS, depending on the experimental design. For the low-serum conditions, cells were primed in serum-free media for 6 h before creating the scratch and treating with media supplemented with 1% or 0.1% FBS and NPY or its receptor antagonists, when desired. Subsequently, the 96-well-plates were placed in the IncuCyte live cell imaging system (Sartorius, Goettingen, Germany) and the images collected every 2 h during the incubation time. The IncuCyte ZOOM software generated a wound width (WW) migration metric, which was used to calculate the migration distance (MD) according to the following formula: MD = (WW_T0_ – WW_Tn_)/2.

### Proliferation Assay

CHO-K1 cells stably transfected with EGFP, Y2R-EGFP, and Y5R-EGFP were cultured in 10% FBS for 24 h in 96-well-plates at a density of 2–2.5 × 10^4^ cells per well. Then, the proliferation was assessed under conditions mimicking these used in the migration assays: 10, 1, or 0.1% FBS. For the low-serum conditions, cells were primed in serum-free medium for 6 h before treatment with media supplemented with 1 or 0.1% FBS and NPY or its receptor antagonists, when desired. The cells were then monitored using the IncuCyte® Live-Cell Analysis System providing images every 2 h during incubation time. Phase object confluence metric measured as a percentage of surface coverage was used to evaluate cellular proliferation.

### RhoA Pull-Down Assay

CHO-K1 cells stably transfected with Y5R-EGFP or SK-N-AS NB cells were incubated in serum-free media for 24 or 72 h, respectively, and then stimulated with 10^–7^ M NPY for 20 min or 10^–6^ M Y5R antagonist for 30 min. Cell lysates were collected, and the levels of active-RhoA were measured using RhoA Pull-down Activation Assay Biochem Kit (Cytoskeleton Inc., Denver, CO) according to the manufacturer's protocol. The band intensities were quantified by Image J software (National Institutes of Health, Bethesda, MD). The levels of active RhoA-GTP were normalized to total RhoA expression.

### Immunocytochemistry

Cells were seeded on 12-mm glass coverslips (2.5 × 10^5^ cells in a 35-mm plate) and cultured for 24 h. Then, the cells were fixed for 10 min using 4% paraformaldehyde and blocked in 1% bovine serum albumin (BSA) for 1 h at RT. The subsequent staining with rabbit monoclonal anti-Y5R antibody (1:250; Abcam, Cambridge, MA; cat # ab133757) was performed without permeabilization to detect the fraction of Y5R present on the cell membrane. Subsequently, the samples were incubated with Alexa Fluor 488-conjugated goat anti-rabbit antibody (1:1,000, Invitrogen; cat # A-11008) for 1 h at RT. Then, the cells were permeabilized and stained with mouse monoclonal anti-RhoA-GTP antibody (1:100; NewEast Biosciences, King of Prussia, PA; cat # NE-26904) overnight at 4°C, followed by incubation with AlexaFluor 594-conjugated donkey anti-mouse antibody (1:1,000, Invitrogen; cat # A-21203) for 1 h at RT. Alternatively, NB cells were co-stained with rabbit monoclonal anti-Y5R (1:100; Abcam; cat # ab133757) and mouse monoclonal anti-ganglioside GD2 (1:100; Santa Cruz Biotechnology, Dallas, TX; cat# sc-53831) antibodies. For actin filament detection, cells were permeabilized and stained with Texas Red®-X phalloidin (1:50, Molecular Probes, Eugene, OR) for 20 min, while DAPI at a concentration of 0.5 μg/ml in PBS was used to detect DNA.

### Proximity Ligation Assay

NB cells were seeded on 12-mm glass coverslips (2.5 × 10^5^ cells) that were placed in a 24-well-plate. On the second day after seeding, cells were incubated in serum-free media for 24 h before stimulation with 10^−8^ M NPY for 20 min, with or without a 30-min pre-incubation with 10^−6^ M Y5R antagonist. The cells were fixed for 10 min using 4% paraformaldehyde followed by permeabilization for 10 min. To test the interaction between Y5R and RhoA-GTP, Duolink® *In situ* Red starter kit Mouse/Rabbit (Sigma-Aldrich) was used according to the manufacture's protocol with the following antibodies: rabbit monoclonal anti-Y5R (1:250; Novus Biologicals; cat # NBP1-00957), mouse monoclonal anti-RhoA-GTP (1:100; NewEast Biosciences; cat # NE-26904), and mouse monoclonal anti-HDAC1 as a negative control (1:50, Santa Cruz Biotechnology; cat# SC-8410). For actin filament detection, Alexa Fluor 488-Phalloidin (1:100; Molecular Probes) was used for 20 min before mounting. Intensities of fluorescent staining in the entire cell colonies, their outer plasma membranes, and inner areas were quantified using Image J software.

### Chemotaxis Assay

CHO-K1 transfectants were seeded in the center of chemotaxis chambers (Ibidi, Martinsried, Planegg, Germany). After cell attachment, 10% FBS media supplemented with NPY (10^−9^-10^−7^ M) was added to the right side chamber, while 10% FBS media alone was inserted to the left side chamber. Images were captured every 20 min for a period of 24 h using live cell microscopy at a magnification of 10×. Manual cell tracking for at least 40 cells per condition was performed using ImageJ, and the data were imported into the Chemotaxis software plugin for ImageJ (Ibidi). The software calculated individual cell velocity, euclidean, and accumulated distances, directionality (euclidean distance/accumulated distance), forward migration index (FMI; *x* or *y* coordinate of endpoint/accumulated distance), center of mass (spatial average of coordinates in *x* or *y* direction), and generated trajectory plots of cell migration. Values for center of mass and forward migration index can be positive or negative, depending on whether migration was toward or away from the chemoattractant, respectively. Rayleigh tests for vector data, which account for cell endpoints and the distance from origin, were used to establish whether cellular migration was random (*p* > 0.05) or directed toward the chemoattractant (*p* < 0.05).

### Filopodia Formation

CHO-K1/Y5R-EGFP, CHO-K1/Y2R-EGFP, CHO-K1/EGFP, and SK-N-AS cells were seeded in two-well inserts (Ibidi). After removal of the insert, cells were cultured in 10% FBS media supplemented with 10^−9^-10^−7^ M NPY or were untreated and then were allowed to migrate for 24 h. After fixation, CHO-K1/Y5R-EGFP, CHO-K1/Y2R-EGFP, and CHO-K1/EGFP cells were permeabilized and phalloidin was added for 2 h (1:50, Sigma Aldrich). SK-N-AS cells were fixed, immunostained for Y5R, and counterstained with Texas Red®-X phalloidin and DAPI, as described above. Twenty-five cells per treatment in all cell lines were randomly selected for quantification of filopodia. Numbers of filopodia per cell were quantified using Image J. The percentages of Y5R-positive filopodia were quantified in SK-N-AS cells.

### Statistical Analyses

Statistical analyses were performed using GraphPad Prism 6 software. Between-group comparisons were assessed using one-way repeated measures ANOVA with *post-hoc t*-test, independent-samples *t*-tests, or paired-samples *t*-tests. For count data, Chi-square tests were used. All experiments were performed at least two times, and the combined data are presented as mean ± standard errors.

## Data Availability Statement

The raw data supporting the conclusions of this article will be made available by the authors, without undue reservation.

## Ethics Statement

The studies involving human participants were reviewed and approved by Georgetown University Institutional Review Board. Written informed consent to participate in this study was provided by the participants' legal guardian/next of kin.

## Author Contributions

NA, LC, GIG, and JK designed experiments. NA, LC, SG, EK, LA, and SZ performed experiments. SG performed histopathological analysis. NA, LC, GIG, and JK analyzed data. NA, LC, GIG, SG, EK, and JK prepared figures and wrote manuscript. All authors contributed to the article and approved the submitted version.

## Conflict of Interest

The authors declare that the research was conducted in the absence of any commercial or financial relationships that could be construed as a potential conflict of interest.

## Supplementary Material

Supplementary Movie 1 CHO-K1/Y5R-EGFP cell migration.

Supplementary Figure 1 CHO-K1 cell proliferation. Proliferation of CHO-K1 cells transfected with EGFP alone, Y2R-EGFP or Y5R-EGFP measured by the InCucyte ZOOM live-cell imaging system. **(A)** Proliferation in 10% FBS. **(B)** Proliferation in 1% FBS. **(C)** Proliferation in 0.1% FBS, with or without NPY (10^−10^-10^−7^ M). ^*^*p* < 0.05 by one-way ANOVA followed by Dunnett's test.

Supplementary Figure 2 Uncropped blots—Y5R expression. Full blot for the data presented in Figure 3A—comparison of Y5R expression in SK-N-AS and SK-N-BE(2) cells. The density of Y5R bands was normalized to the density of the unspecific protein staining in the entire lane.

Supplementary Figure 3 Uncropped blots—RhoA pull down experiments. **(A)** Full blot for the data presented in Figure 8B—NPY-induced RhoA activation in CHO-K1/Y5R-EGFP. **(B)** Full blot for the data presented in Figure 8C—NPY dose response in SK-N-AS cells. Both RhoA-GTP and total RhoA were detected with anti-RhoA antibody. His-RhoA served as a Western blot positive control.

Supplementary Figure 4 PLA negative controls in SK-N-AS cells. **(A)** Negative controls for the data presented in Figure 9A—time course of the response to NPY. PLA signal with single antibodies—anti-Y5R or anti-RhoA-GTP. **(B)** Negative controls for the data presented in Figures 9B,C. PLA (red) with single anti-Y5R or anti-RhoA-GTP and non-relevant anti-HDAC1antibodies in SK-N-AS cells stained with phalloidin (green).
