# Supplementary figures and images for: Neuropeptide Y/Y5 Receptor Pathway Stimulates Neuroblastoma Cell Motility Through RhoA Activation
Source: Front Cell Dev Biol. 2021 Feb 17;8:627090. doi: 10.3389/fcell.2020.627090 (PMC7928066; doi:10.3389/fcell.2020.627090)

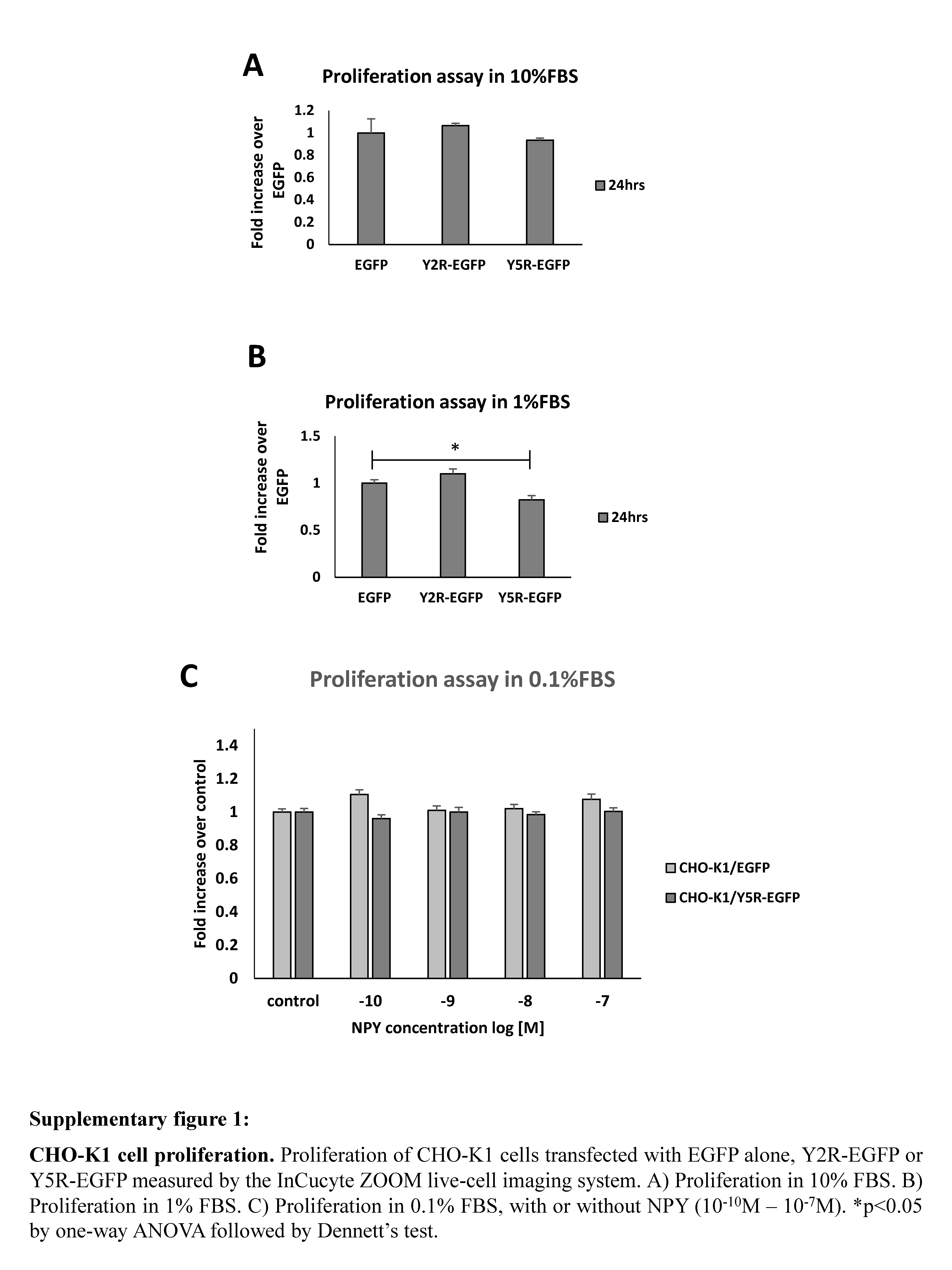

Supplement: Supplementary Figure 1 — CHO-K1 cell proliferation. Proliferation of CHO-K1 cells transfected with EGFP alone, Y2R-EGFP or Y5R-EGFP measured by the InCucyte ZOOM live-cell imaging system. (A) Proliferation in 10% FBS. (B) Proliferation in 1% FBS. (C) Proliferation in 0.1% FBS, with or without NPY (10−10-10−7 M). *p < 0.05 by one-way ANOVA followed by Dunnett's test. [file Image_1.TIF]

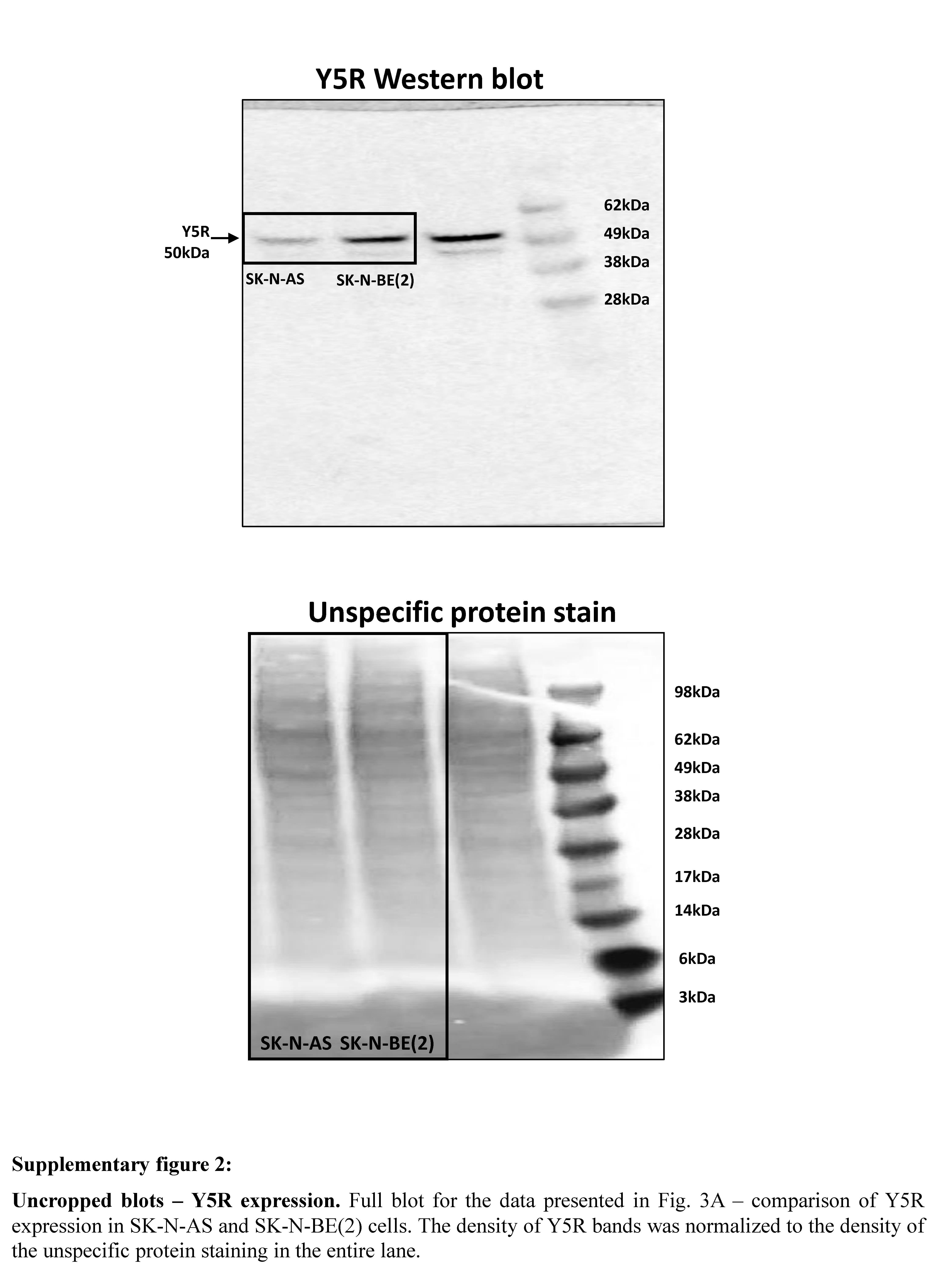

Supplement: Supplementary Figure 2 — Uncropped blots—Y5R expression. Full blot for the data presented in Figure 3A—comparison of Y5R expression in SK-N-AS and SK-N-BE(2) cells. The density of Y5R bands was normalized to the density of the unspecific protein staining in the entire lane. [file Image_2.TIF]

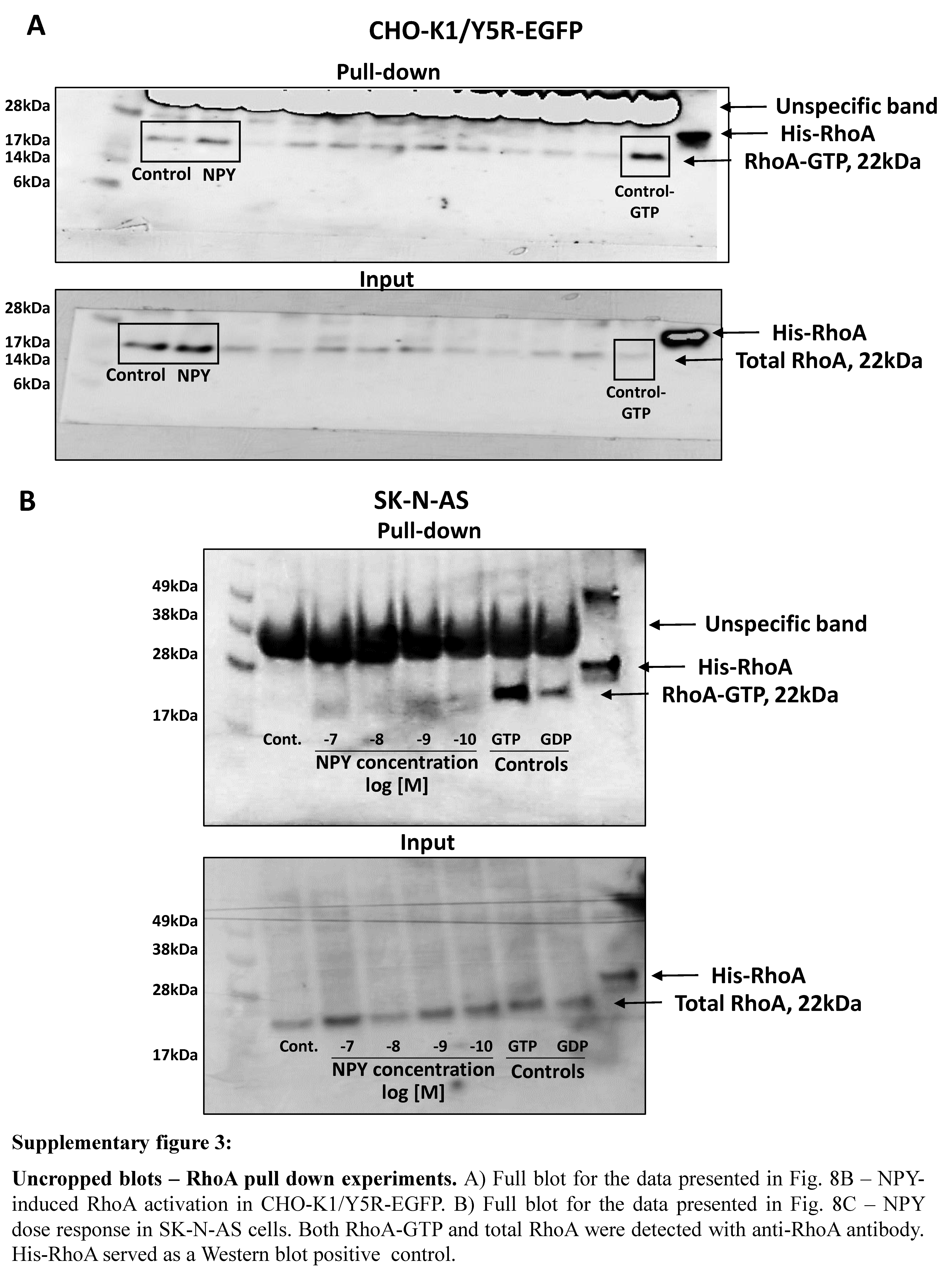

Supplement: Supplementary Figure 3 — Uncropped blots—RhoA pull down experiments. (A) Full blot for the data presented in Figure 8B—NPY-induced RhoA activation in CHO-K1/Y5R-EGFP. (B) Full blot for the data presented in Figure 8C—NPY dose response in SK-N-AS cells. Both RhoA-GTP and total RhoA were detected with anti-RhoA antibody. His-RhoA served as a Western blot positive control. [file Image_3.TIF]

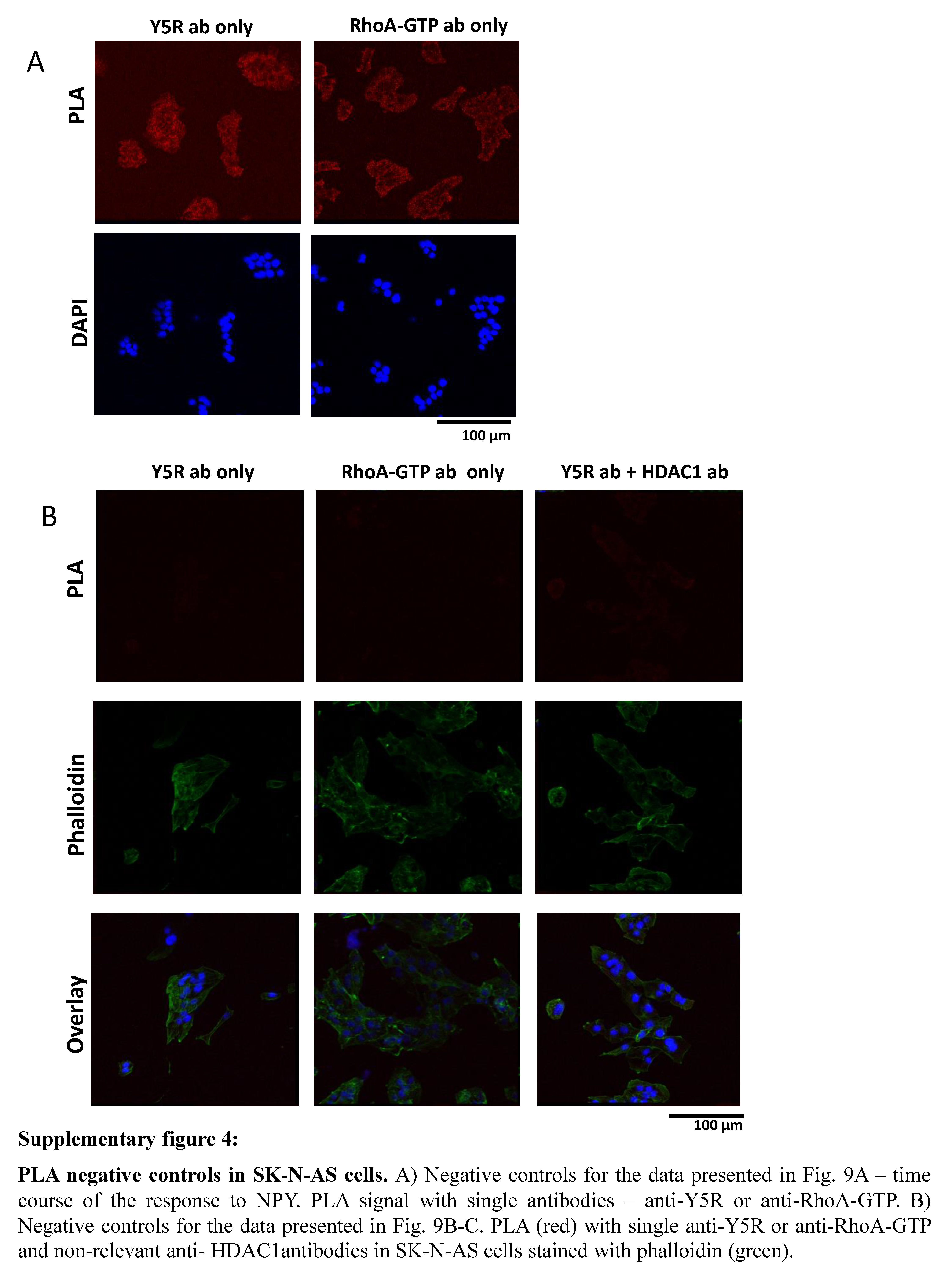

Supplement: Supplementary Figure 4 — PLA negative controls in SK-N-AS cells. (A) Negative controls for the data presented in Figure 9A—time course of the response to NPY. PLA signal with single antibodies—anti-Y5R or anti-RhoA-GTP. (B) Negative controls for the data presented in Figures 9B,C. PLA (red) with single anti-Y5R or anti-RhoA-GTP and non-relevant anti-HDAC1antibodies in SK-N-AS cells stained with phalloidin (green). [file Image_4.TIF]
